# Supplementary figures and images for: Plant-specific cochaperone SSR1 affects root elongation by modulating the mitochondrial iron-sulfur cluster assembly machinery
Source: PLoS Genet. 2025 Feb 5;21(2):e1011597. doi: 10.1371/journal.pgen.1011597 (PMC11835332; doi:10.1371/journal.pgen.1011597)

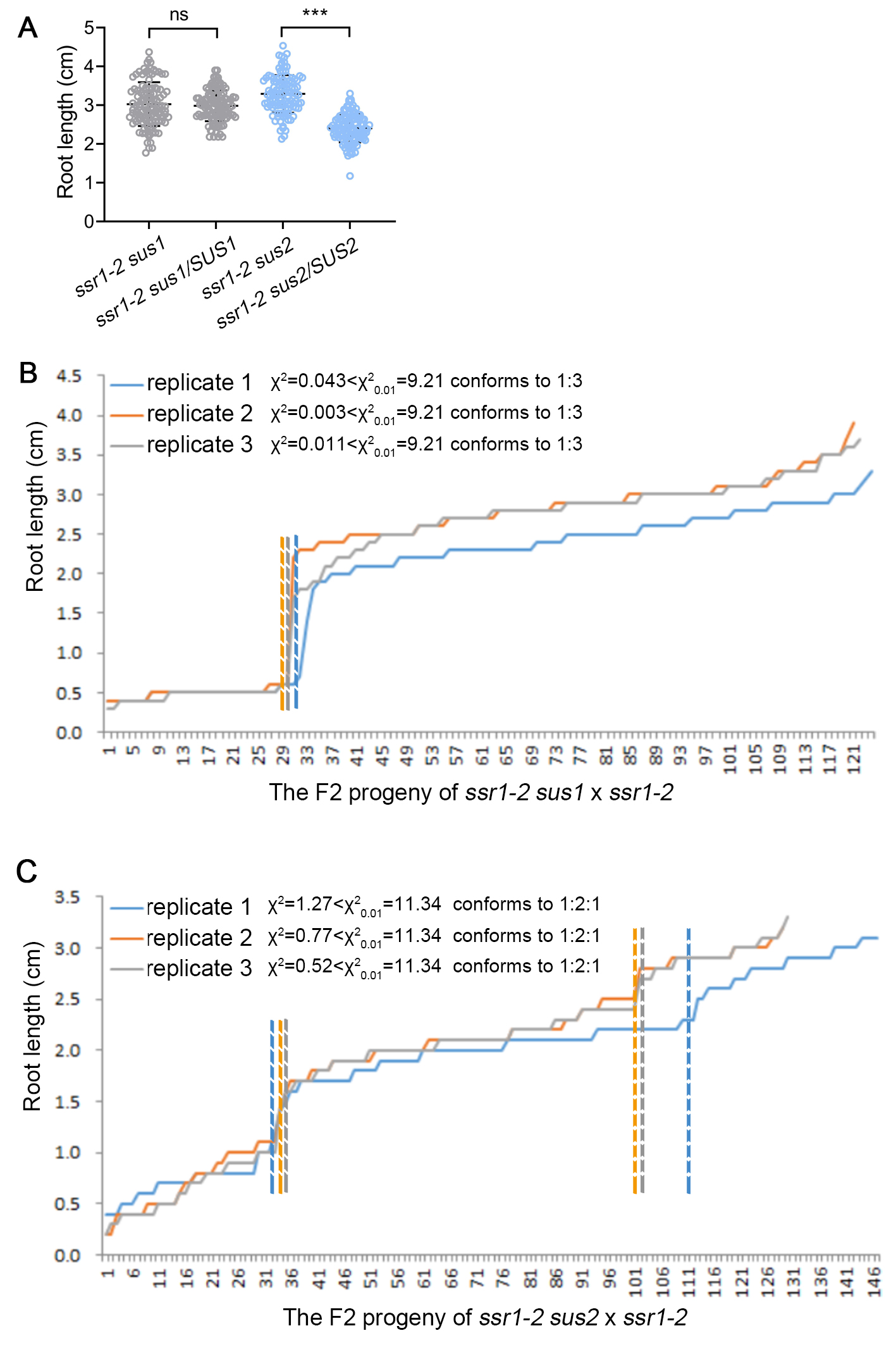

Supplement: S1 Fig — (A) The root length of the F1 population of ssr1-2 sus1 x ssr1-2 and ssr1-2 sus2 x ssr1-2 was compared with that of their respective parents, ssr1-2 sus1 and ssr1-2 sus2. (B) The two F1 populations were self-pollinated to generate F2 seeds. The primary root length of the F2 seedlings (10-days-old) were measured. The original root length data are shown in S1 Table. Three biological repeats were performed and shown in differently colored lines. The segregation ratio of the root length phenotype indicates that sus1 is a dominant mutation and sus2 is a semi-dominant mutation. (TIF) [file pgen.1011597.s010.tif]

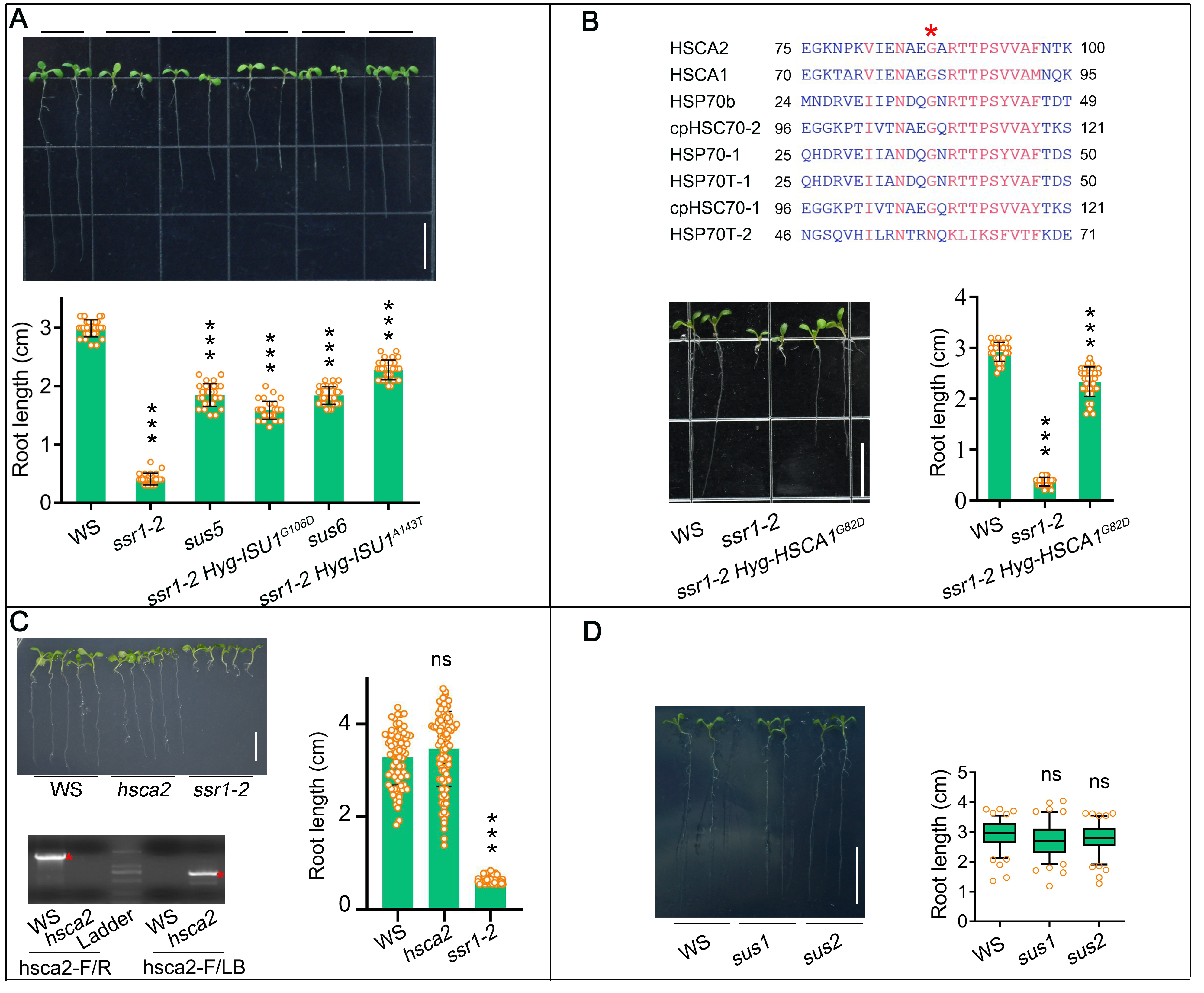

Supplement: S2 Fig — (A) sus5 and sus6 mutant genes were cloned, designated as ISU1G106D and ISU1A143T, respectively, and re-transformed back to ssr1-2 plants. In transgenic lines, the transgenes are associated with a hygromycin resistant gene and therefore designated as Hyg-ISU1G106D or Hyg-ISU1A143T. It should be noted that ssr1-2 Hyg-ISU1G106D and ssr1-2 Hyg-ISU1A143T still contain the wild type ISU1 allele. (B) HSCA1G82D is a homolog of HSCA2G87D, and it can rescue the phenotypes of ssr1-2. The naming rule of transgenic line was similar to 1A. The red asterisk labeled amino acid residue G is conserved among various member of HSP70 family except HSP70T-2. (C) HSCA2 loss of function mutation has no effect on root length. Primers hsca2-F and hsca2-R were used for the amplification of HSCA2 genome fragment, and hsca2-F and LB were used to detect the T-DNA insertion. (D) The single mutant sus1 and sus2 have similar root length with the wild type. sus1 and sus2 single mutants were isolated after back-crossing ssr1-2 sus1 and ssr1-2 sus2 with the wild-type. For (A-D), representative seedlings grown for 10 days and average root length of analyzed lines were shown. Error bars represent standard deviation from 20 (A), 30 (B), 60 (C) and 100 (D) seedlings on primary roots. Scale bars = 1 cm. The statistical significance of the results was determined through the use of a student t-test. Variations were considered significant if P <0.05(*), 0.01(**) or 0.001(***). (TIF) [file pgen.1011597.s011.tif]

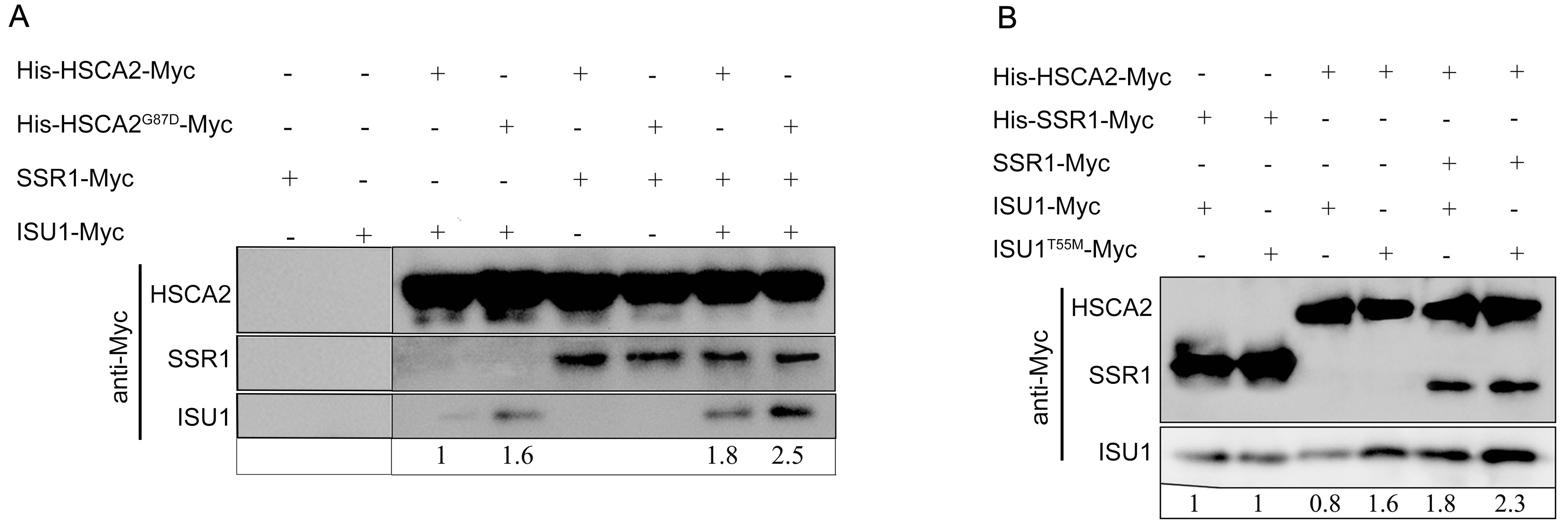

Supplement: S3 Fig — In vitro pull-down assays were conducted using Ni-NTA Sepharose with His-tagged proteins expressed and purified from E. coli as baits. The recombinant proteins SSR1-Myc, ISU1-Myc and ISU1T55M-Myc were expressed in E. coli and the protein lysate was directly used for co-incubation with His-tagged proteins. Co-purified proteins were detected with anti-Myc antibody. The immunoblotting signals of ISU1-Myc or ISU1T55M-Myc were quantified by ImageJ and relative intensities were shown under respective lanes. The mitochondrial targeting peptide coding sequences of HSCA2 and ISU1 were removed for protein expression in E. coli. (TIF) [file pgen.1011597.s012.tif]

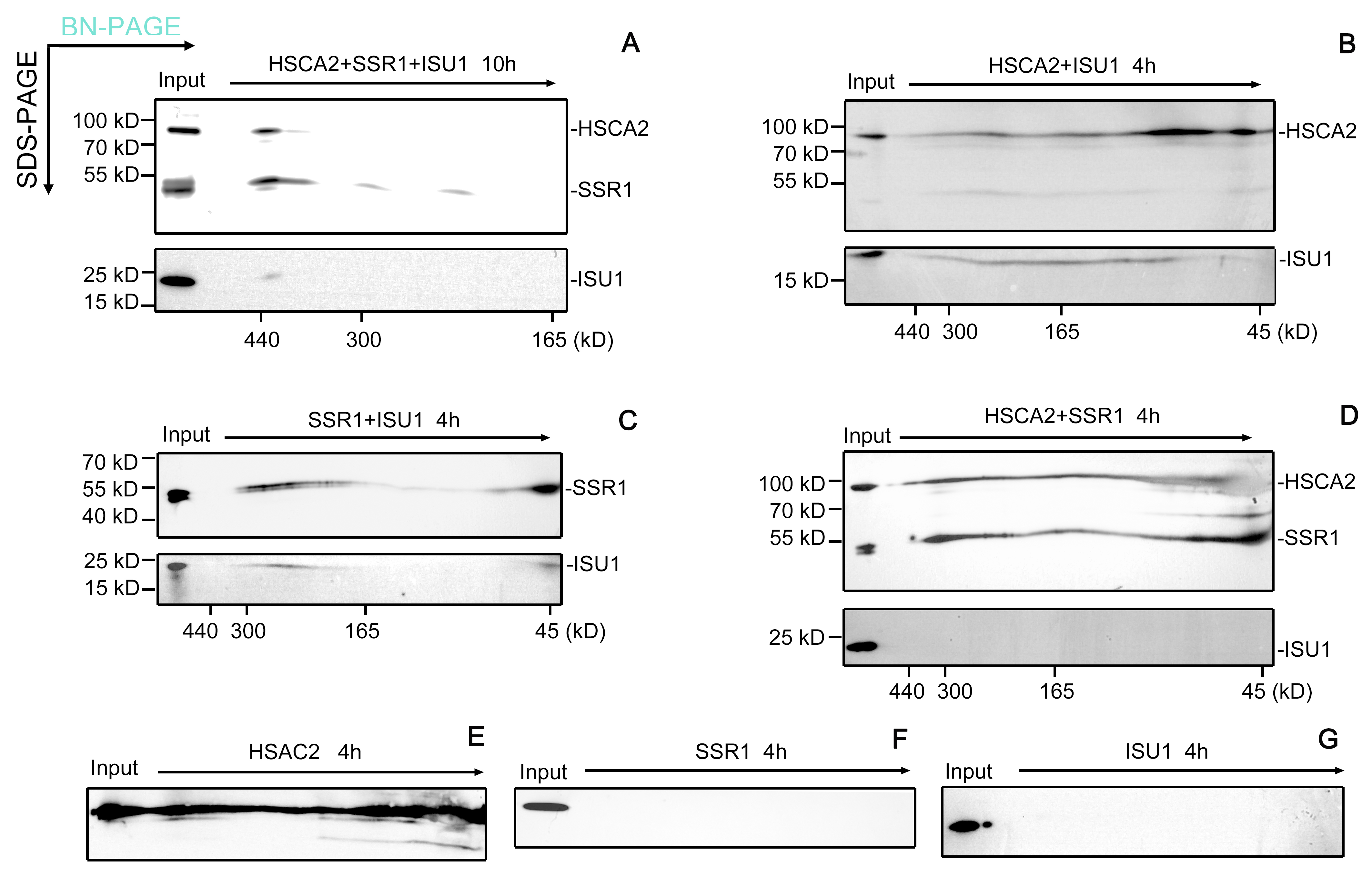

Supplement: S4 Fig — Different designated combinations of proteins were incubated at 4°C for one hour, after which they were subjected to a BN-PAGE assay. Subsequently, the BN-PAGE lanes were incised and rotated to a 90-degree angle for SDS-PAGE assay. Finally, the signal of each protein was detected by anti-Myc antibody. The three proteins could be distinguished based on their respective band sizes. The mitochondrial targeting peptide coding sequences of HSCA2, SSR1 and ISU1 were removed for protein expression in E. coli. (TIF) [file pgen.1011597.s013.tif]

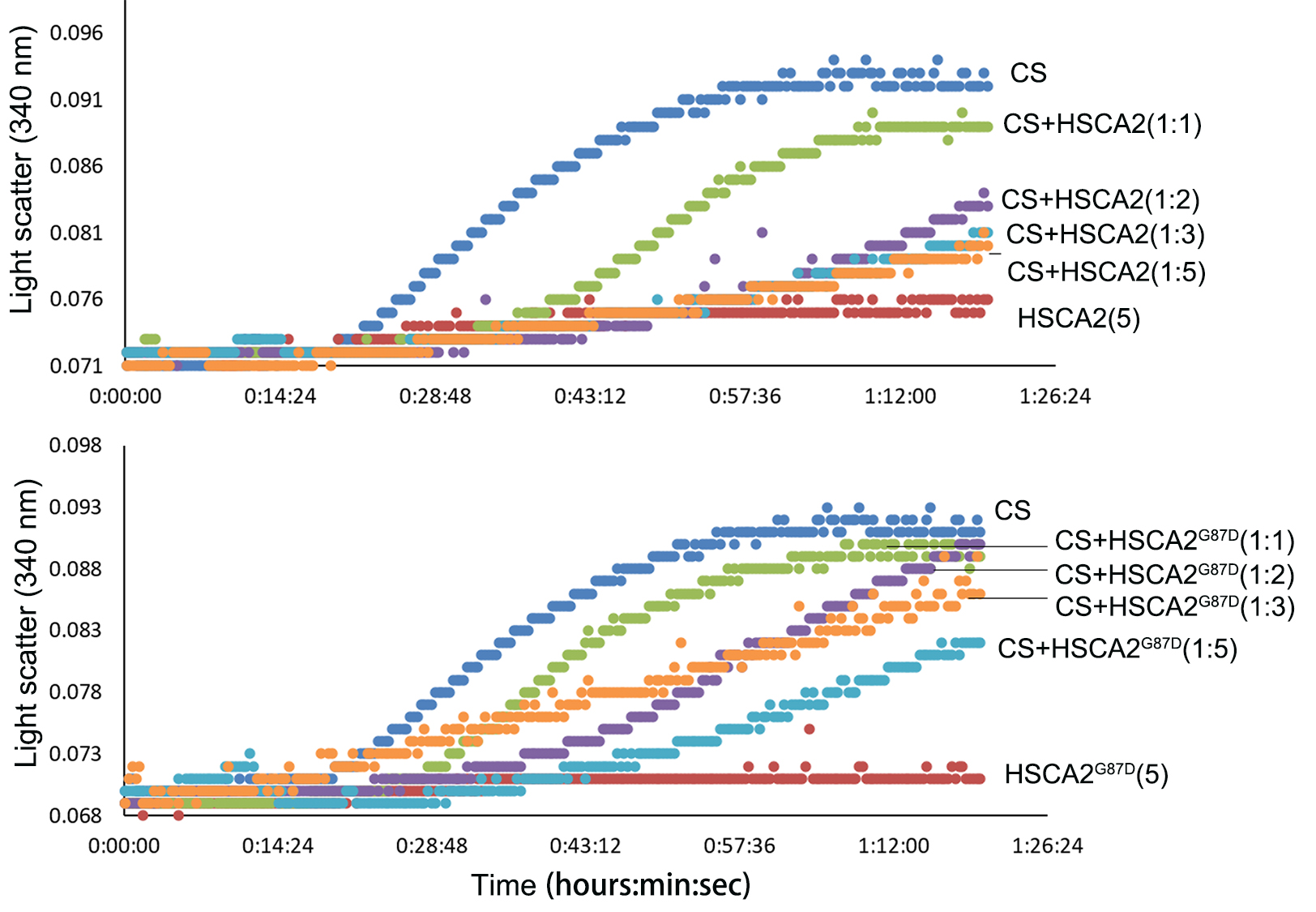

Supplement: S5 Fig — Heat-induced aggregation of citrate synthase (CS) was performed at 45°C for 90min with different amount of purified test proteins. The molecular ratios of CS to tested proteins are indicated following each protein sample. (TIF) [file pgen.1011597.s014.tif]

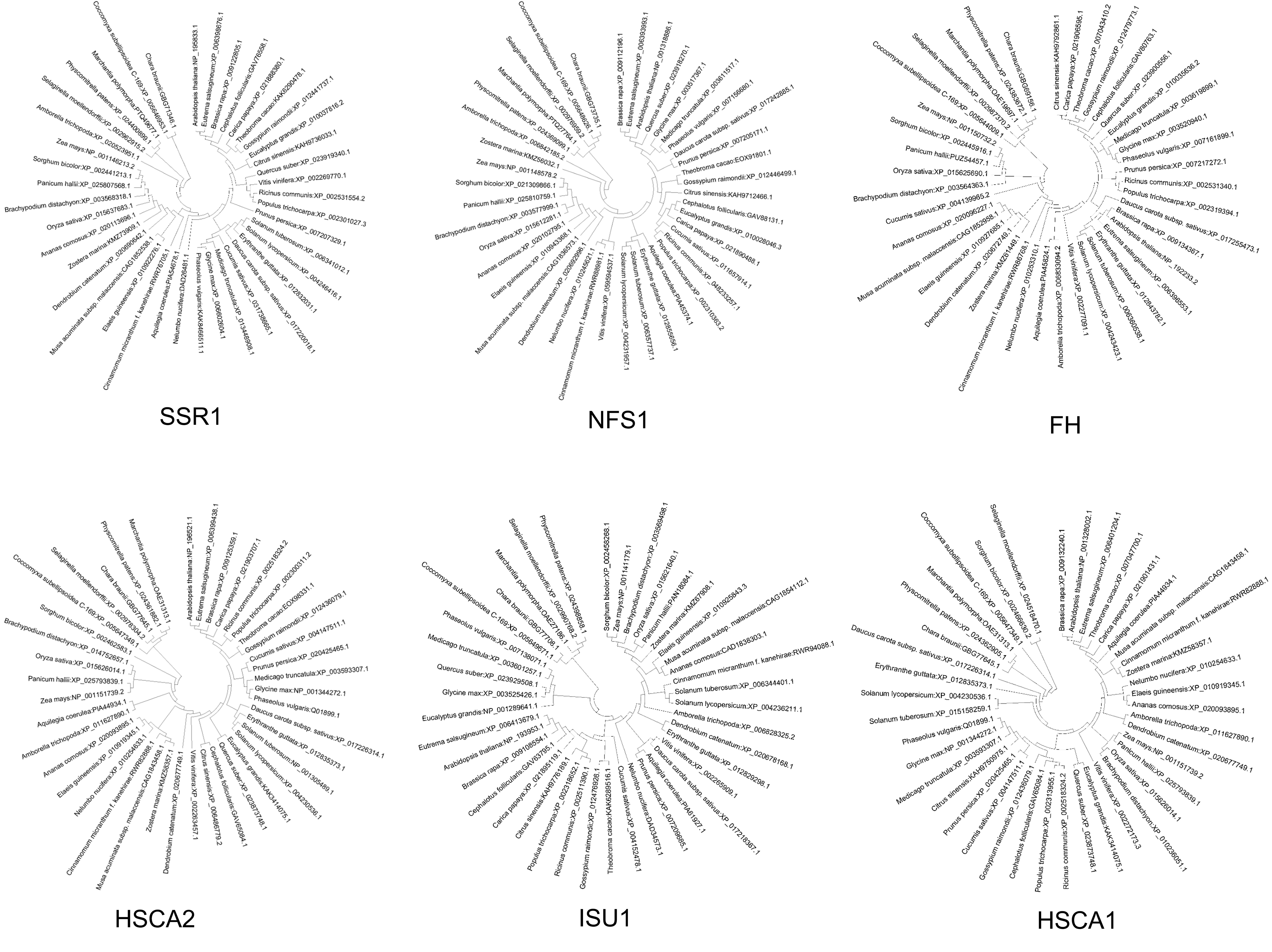

Supplement: S6 Fig — Maximum Likelihood phylogenetic trees of SSR1 orthologs, NFS1 orthologs, FH orthologs, HSCA2 orthologs, ISU1 orthologs and HSCA1 orthologs were constructed using MEGA 7.0.26 with parameters of the Jones-Taylor-Thornton (JTT) model, complete deletion and 1,000 replicates of bootstrap. The species and the accession numbers of sequences are shown in figure and listed in S6 Table. (TIF) [file pgen.1011597.s015.tif]

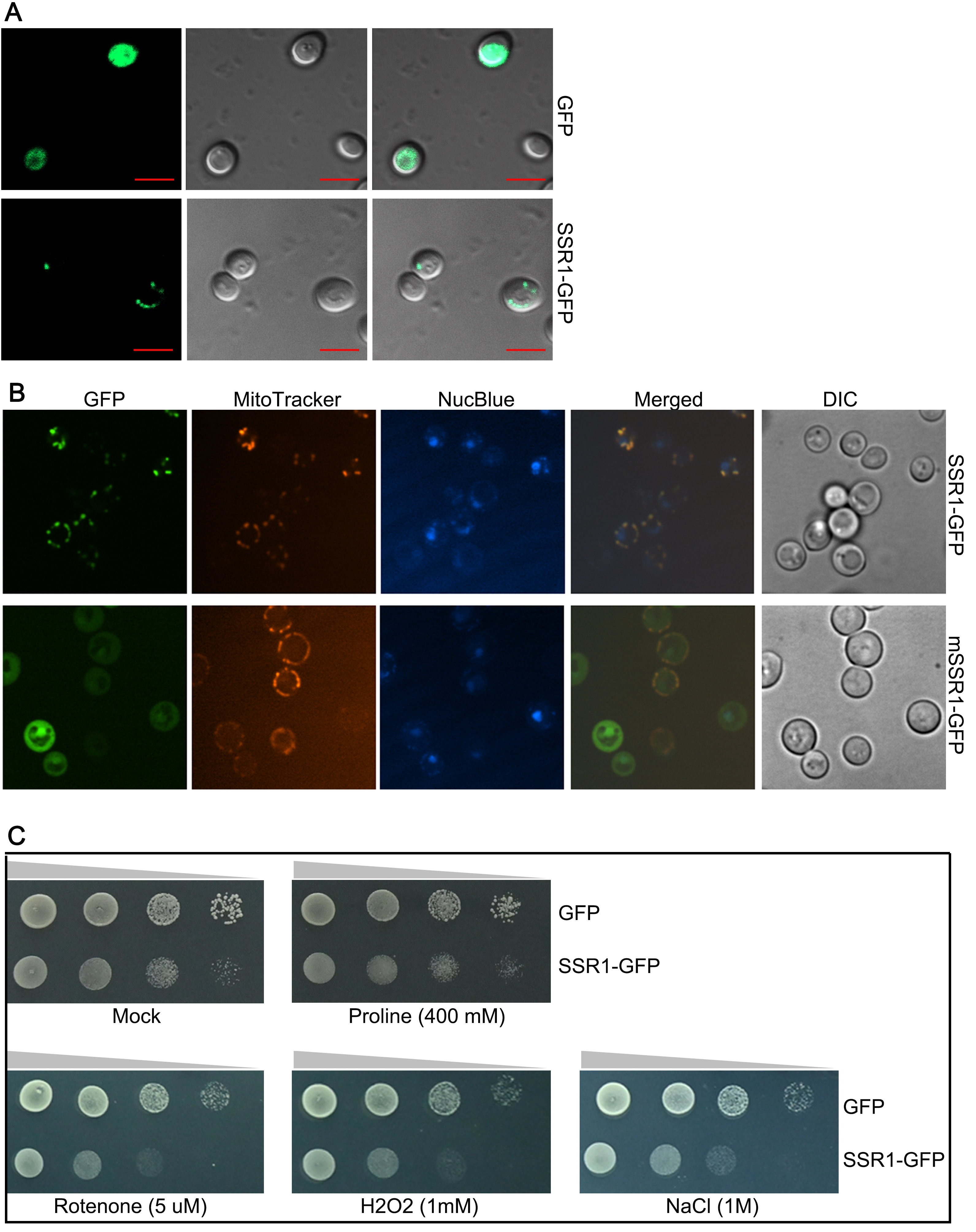

Supplement: S7 Fig — (A) Laser confocal microscopy was employed to observe the expression of GFP and SSR1-GFP in yeast cells. (B) The yeast cells expressing GFP or SSR1-GFP were exposed to different stress conditions. (C) Subcellular localization of SSR1-GFP and mSSR1-GFP in yeast cells. mSSR1 represents the deletion of the N-terminal mitochondrial localization signal peptide. Scale bars represent 5 μm. (TIF) [file pgen.1011597.s016.tif]
