# Supplementary material for: Plant-specific cochaperone SSR1 affects root elongation by modulating the mitochondrial iron-sulfur cluster assembly machinery
Source: PLoS Genet. 2025 Feb 5;21(2):e1011597. doi: 10.1371/journal.pgen.1011597 (PMC11835332; doi:10.1371/journal.pgen.1011597)
Supplement: S2 Appendix — (PPTX) [file pgen.1011597.s019.pptx]

## Slide 1
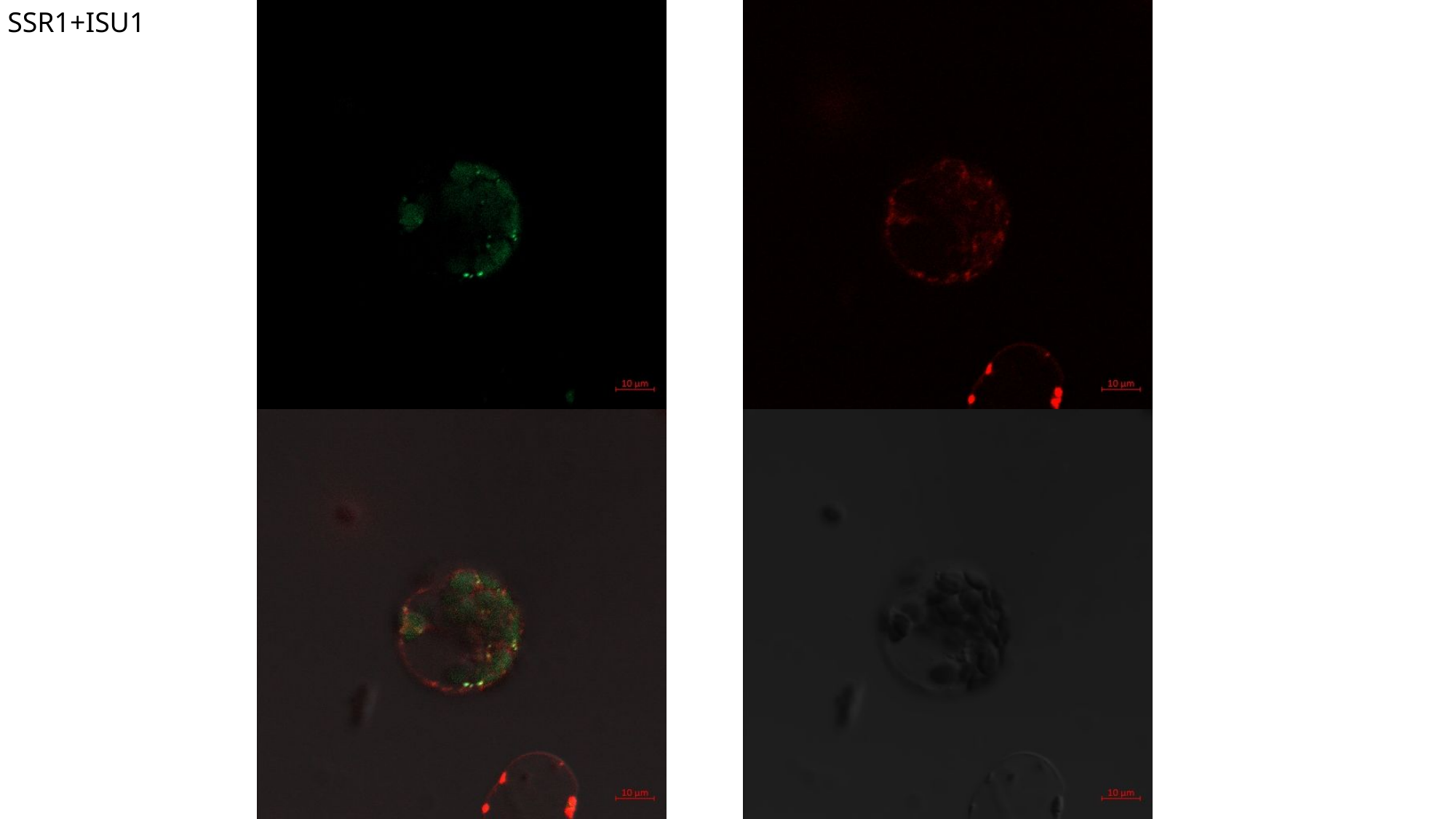

SSR1+ISU1

## Slide 2
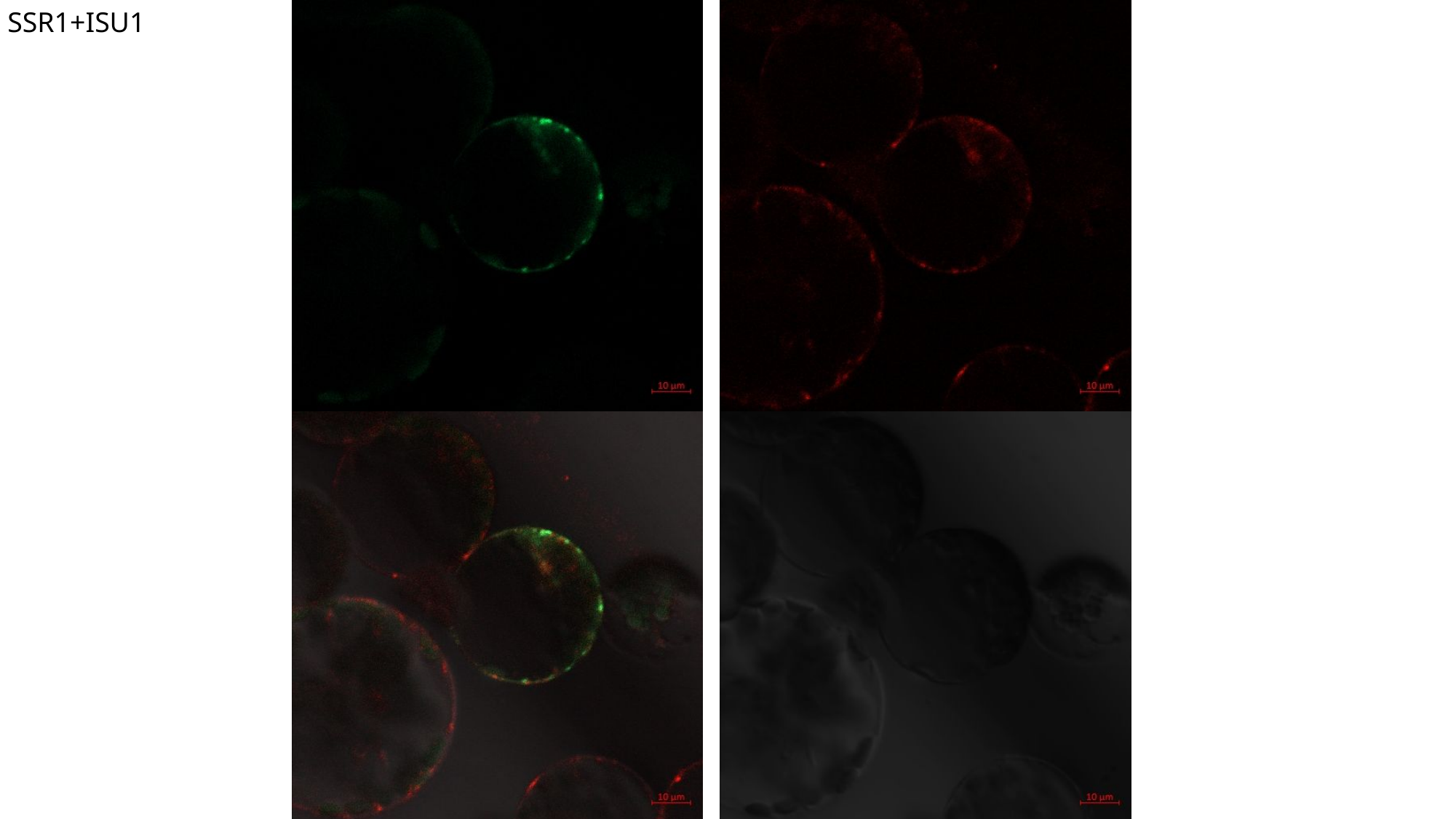

SSR1+ISU1

## Slide 3
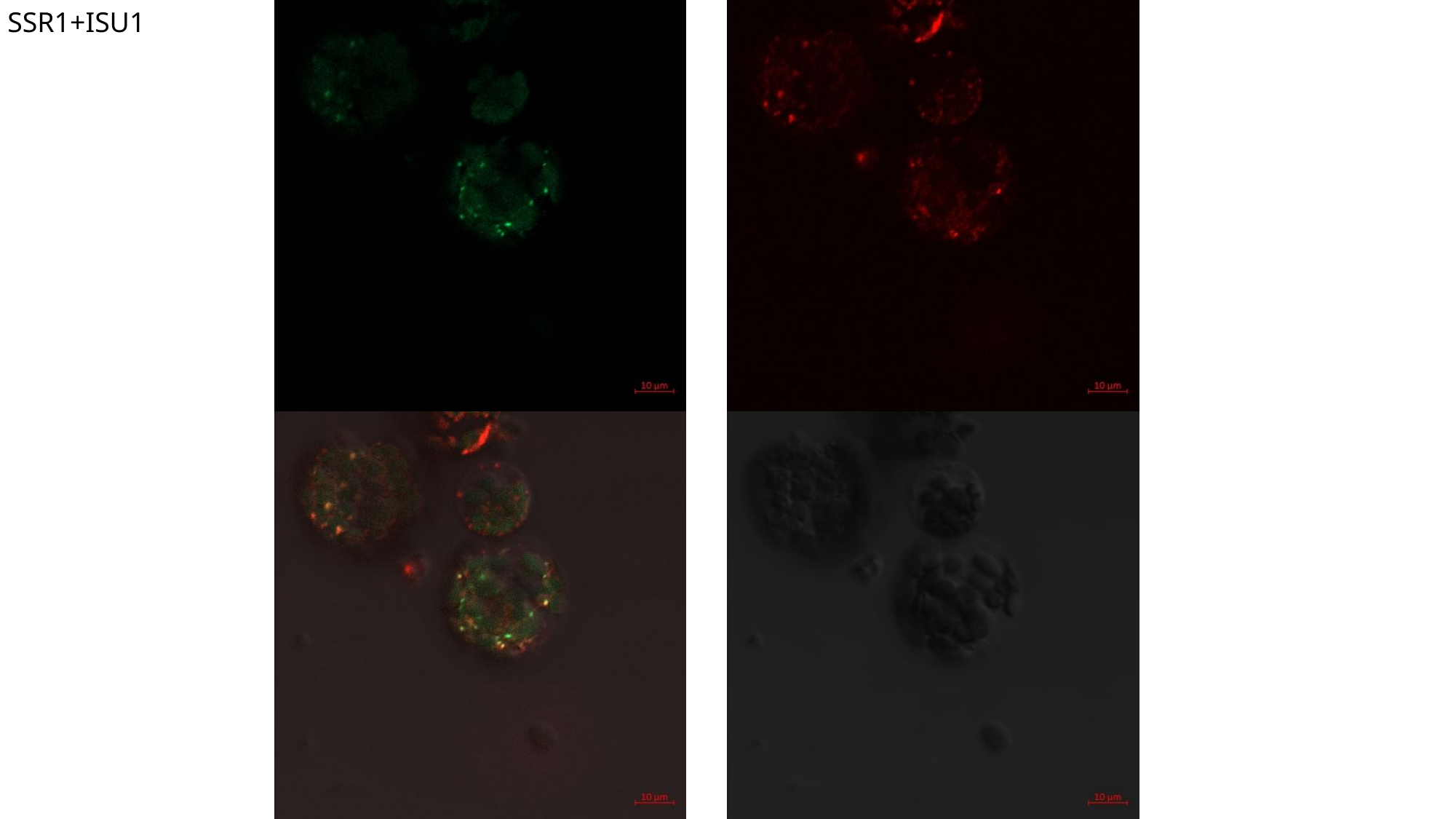

SSR1+ISU1

## Slide 4
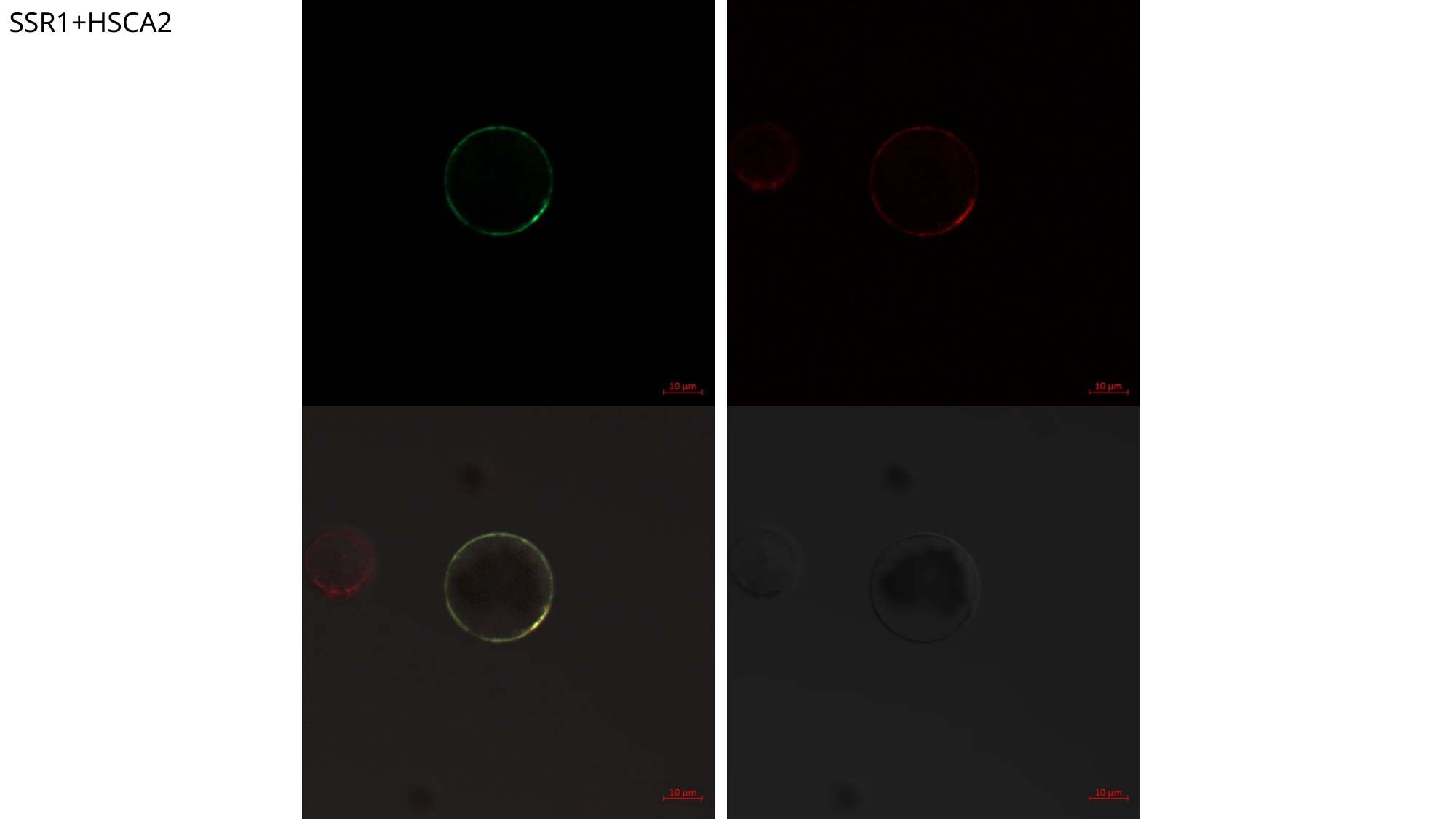

SSR1+HSCA2

## Slide 5
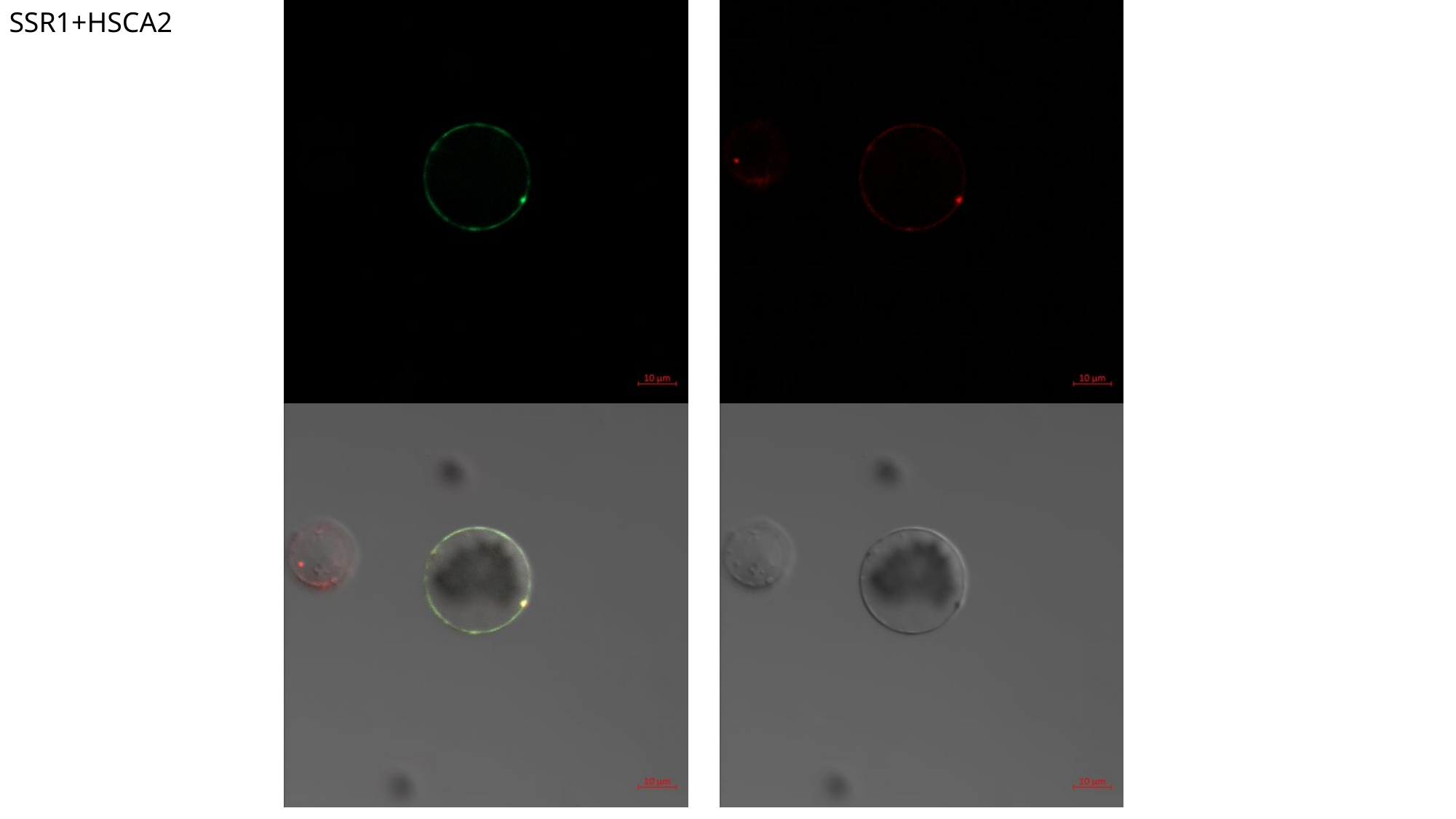

SSR1+HSCA2

## Slide 6
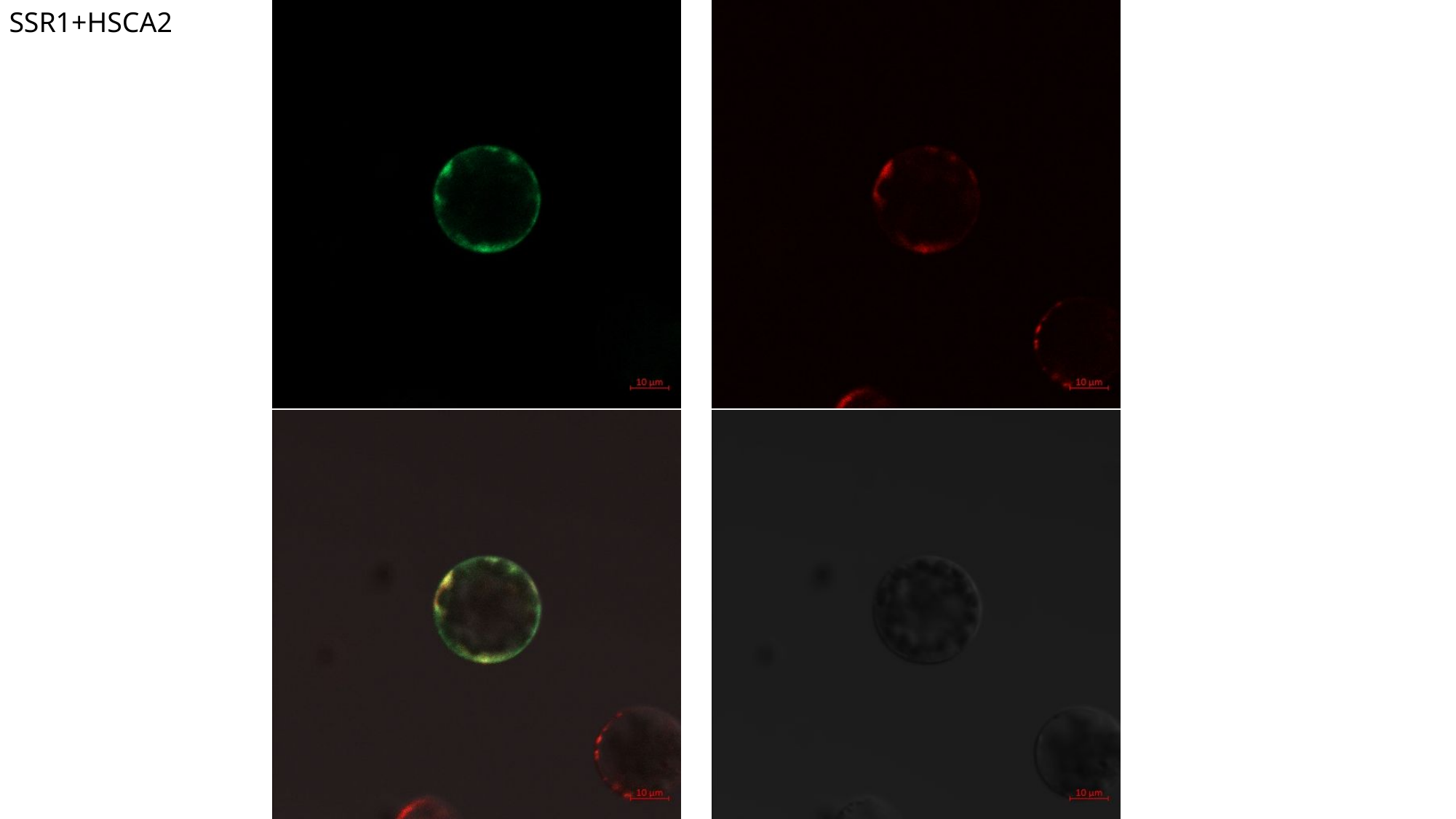

SSR1+HSCA2

## Slide 7
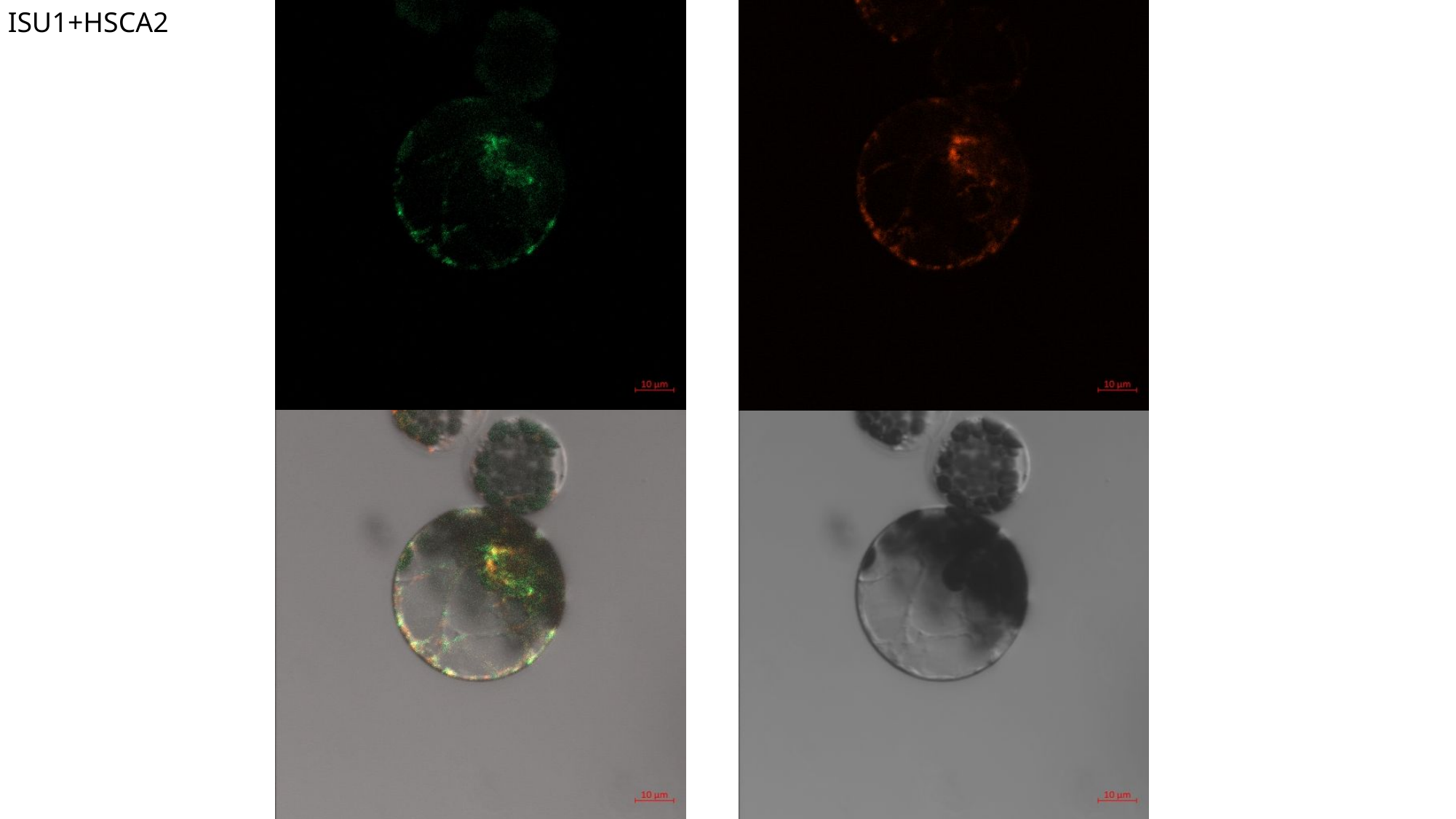

ISU1+HSCA2

## Slide 8
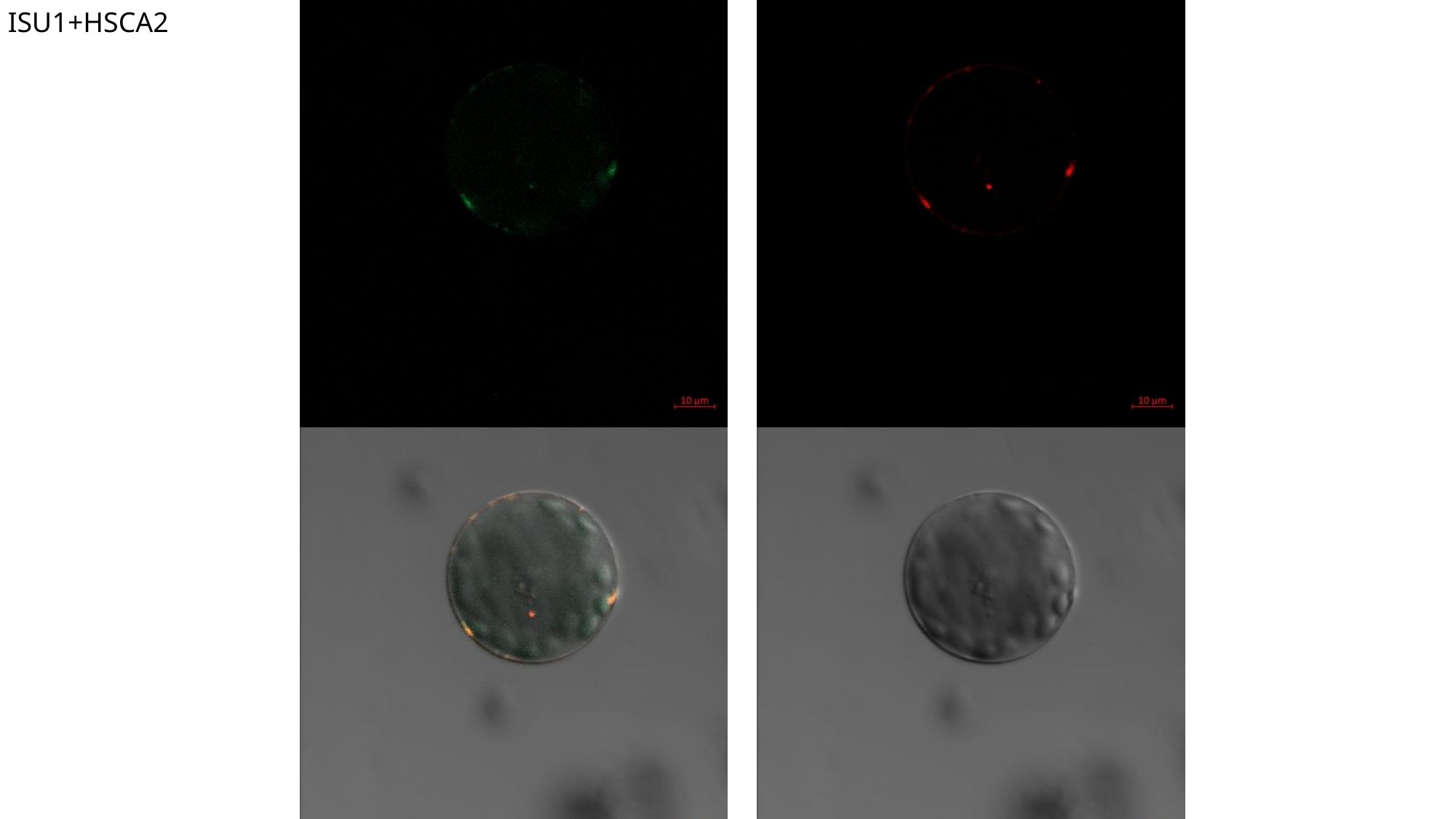

ISU1+HSCA2

## Slide 9
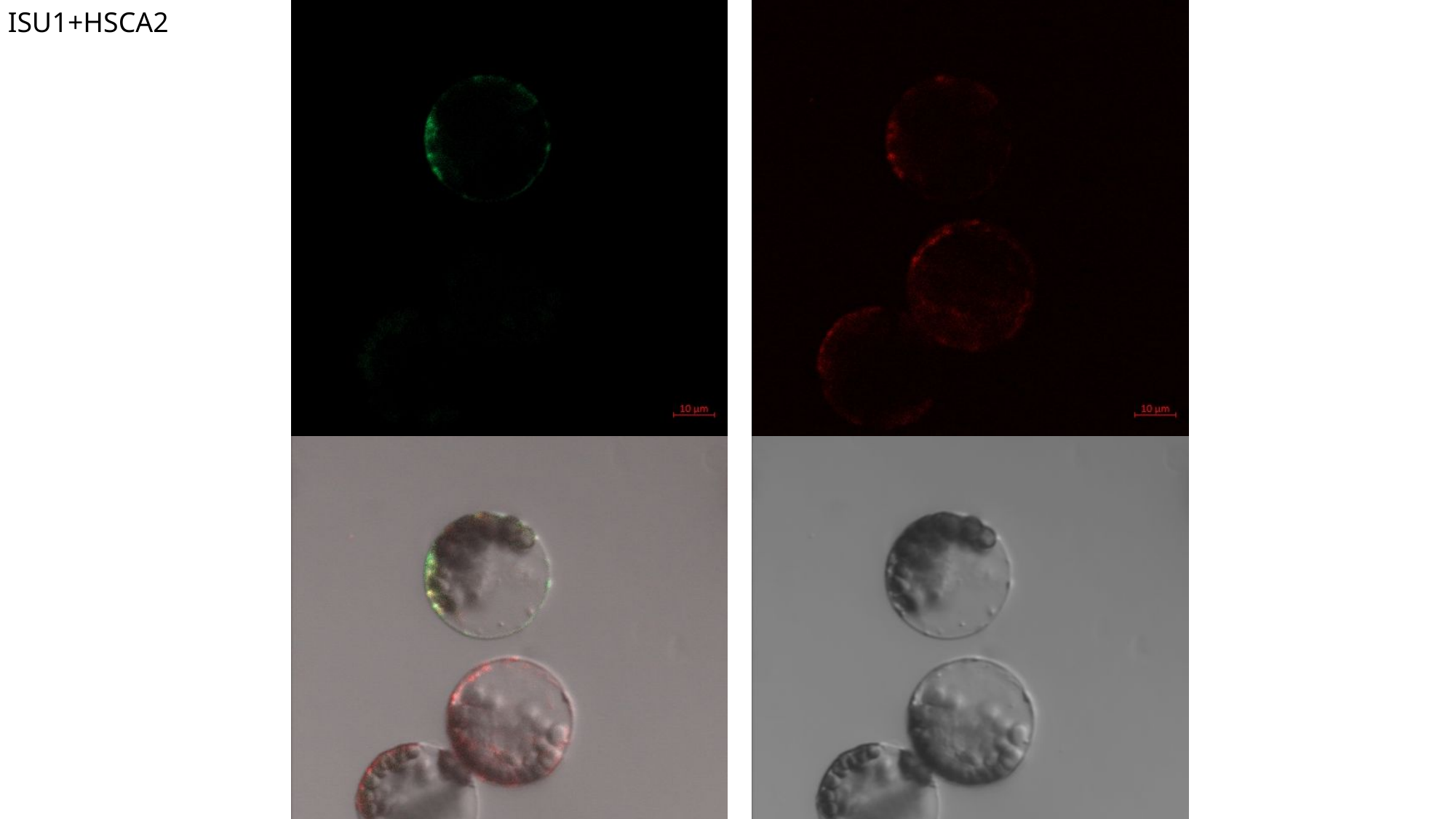

ISU1+HSCA2
