# Supplementary material for: Plant-specific cochaperone SSR1 affects root elongation by modulating the mitochondrial iron-sulfur cluster assembly machinery
Source: PLoS Genet. 2025 Feb 5;21(2):e1011597. doi: 10.1371/journal.pgen.1011597 (PMC11835332; doi:10.1371/journal.pgen.1011597)
Supplement: S3 Appendix — (PPTX) [file pgen.1011597.s020.pptx]

## Slide 1
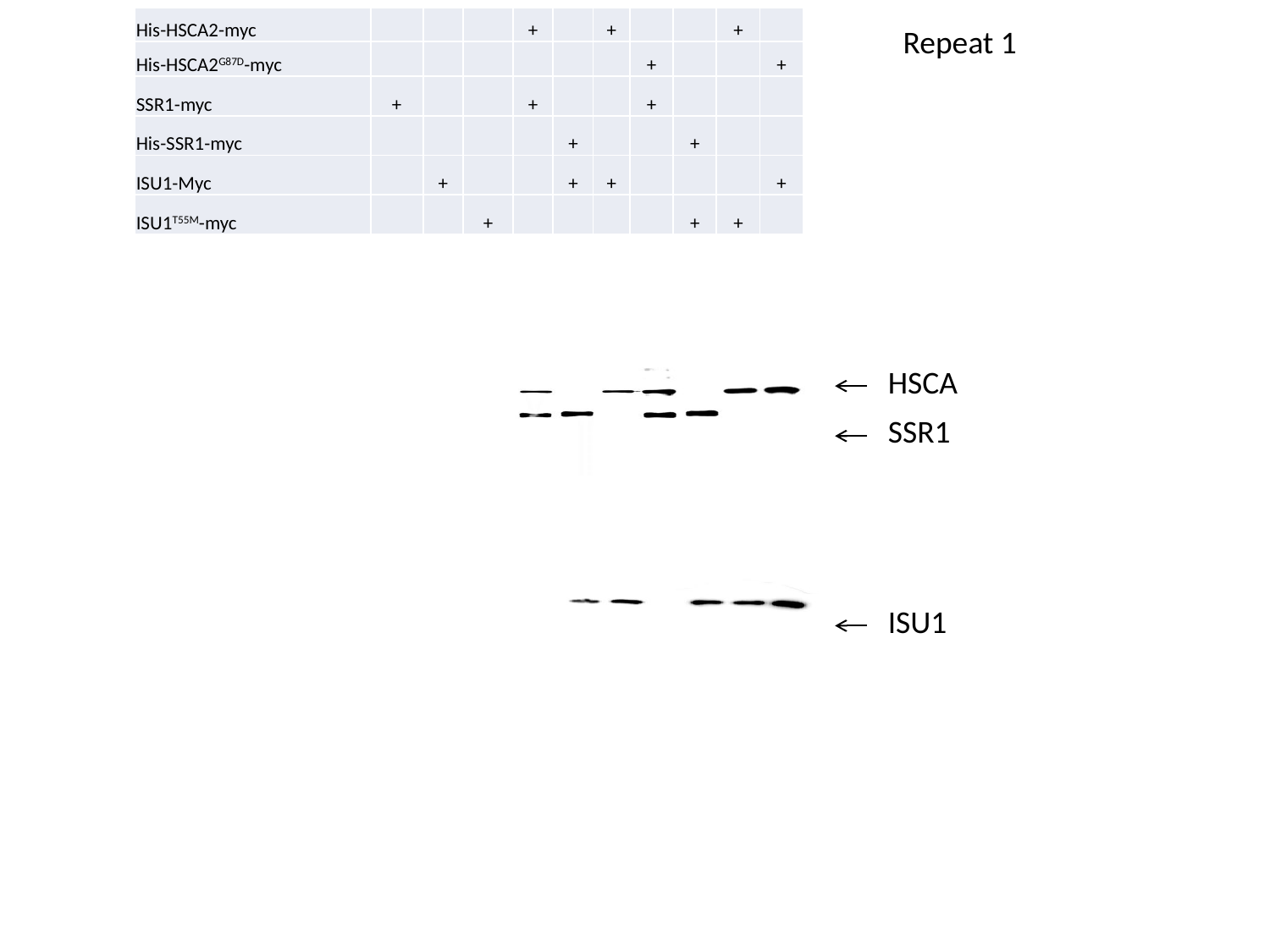

| His-HSCA2-myc | | | | + | | + | | | + | |
| --- | --- | --- | --- | --- | --- | --- | --- | --- | --- | --- |
| His-HSCA2G87D-myc | | | | | | | + | | | + |
| SSR1-myc | + | | | + | | | + | | | |
| His-SSR1-myc | | | | | + | | | + | | |
| ISU1-Myc | | + | | | + | + | | | | + |
| ISU1T55M-myc | | | + | | | | | + | + | |
Repeat 1
HSCA
SSR1
ISU1

## Slide 2
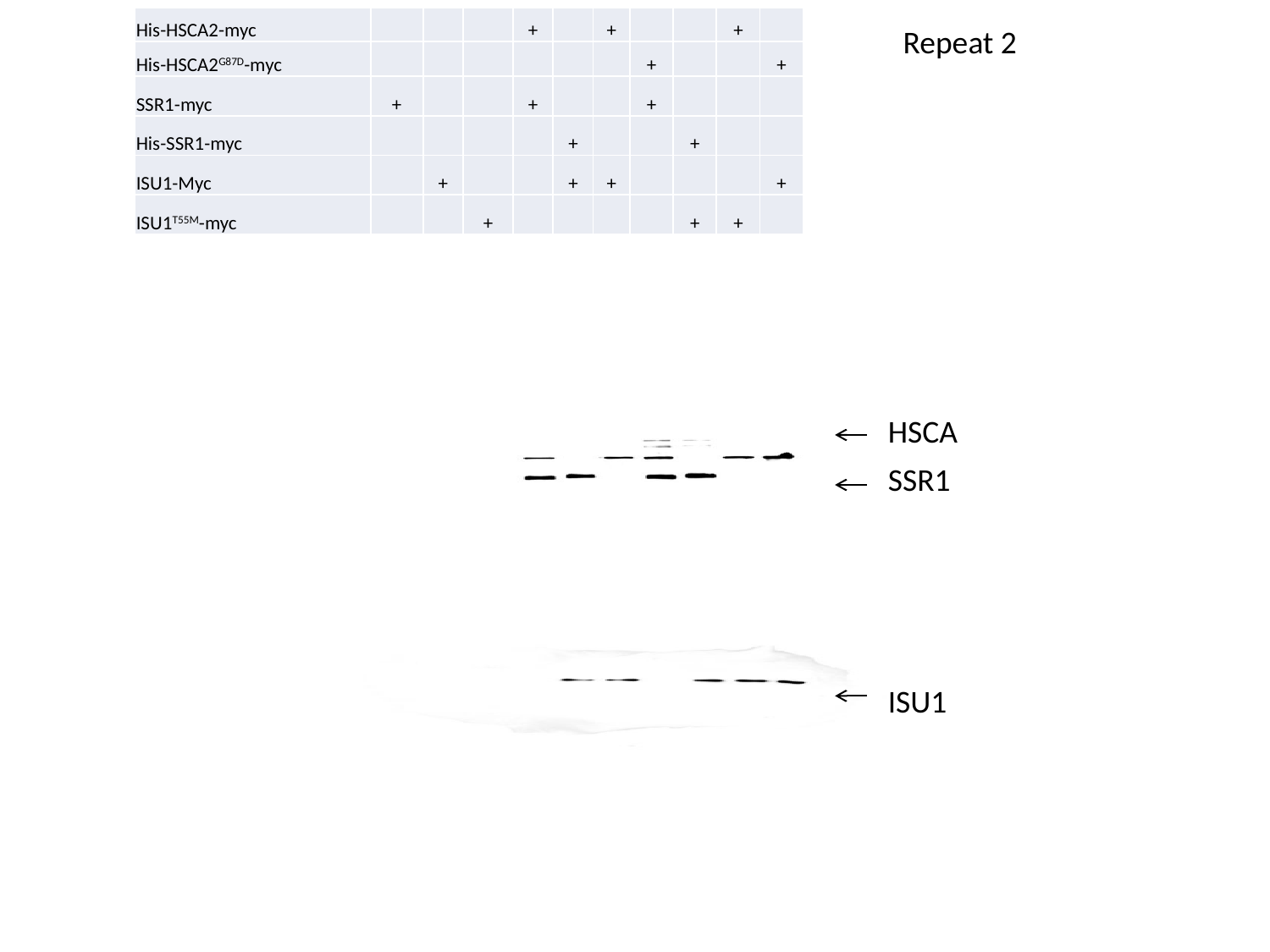

| His-HSCA2-myc | | | | + | | + | | | + | |
| --- | --- | --- | --- | --- | --- | --- | --- | --- | --- | --- |
| His-HSCA2G87D-myc | | | | | | | + | | | + |
| SSR1-myc | + | | | + | | | + | | | |
| His-SSR1-myc | | | | | + | | | + | | |
| ISU1-Myc | | + | | | + | + | | | | + |
| ISU1T55M-myc | | | + | | | | | + | + | |
Repeat 2
HSCA
SSR1
ISU1

## Slide 3
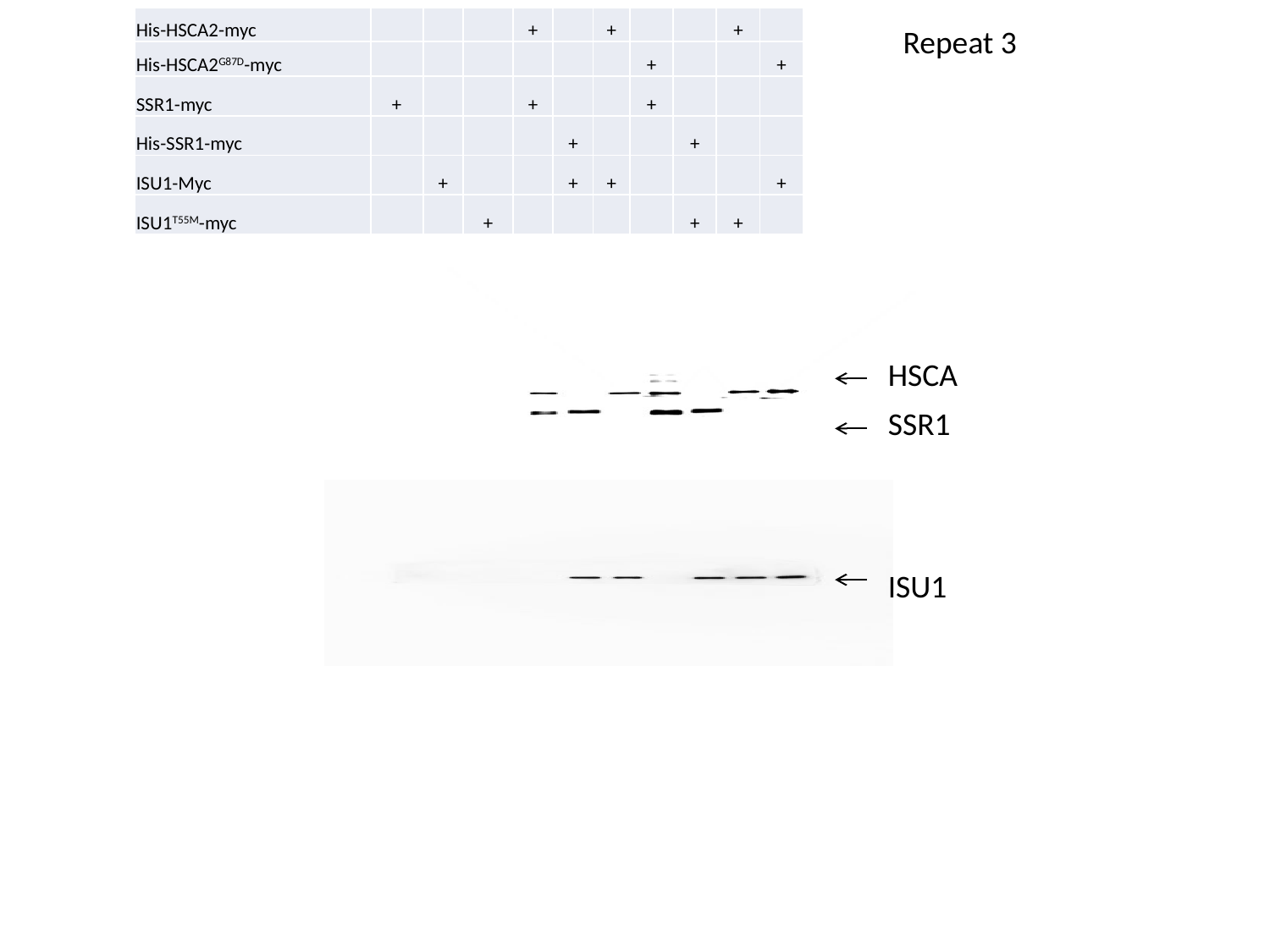

| His-HSCA2-myc | | | | + | | + | | | + | |
| --- | --- | --- | --- | --- | --- | --- | --- | --- | --- | --- |
| His-HSCA2G87D-myc | | | | | | | + | | | + |
| SSR1-myc | + | | | + | | | + | | | |
| His-SSR1-myc | | | | | + | | | + | | |
| ISU1-Myc | | + | | | + | + | | | | + |
| ISU1T55M-myc | | | + | | | | | + | + | |
Repeat 3
HSCA
SSR1
ISU1

## Slide 4
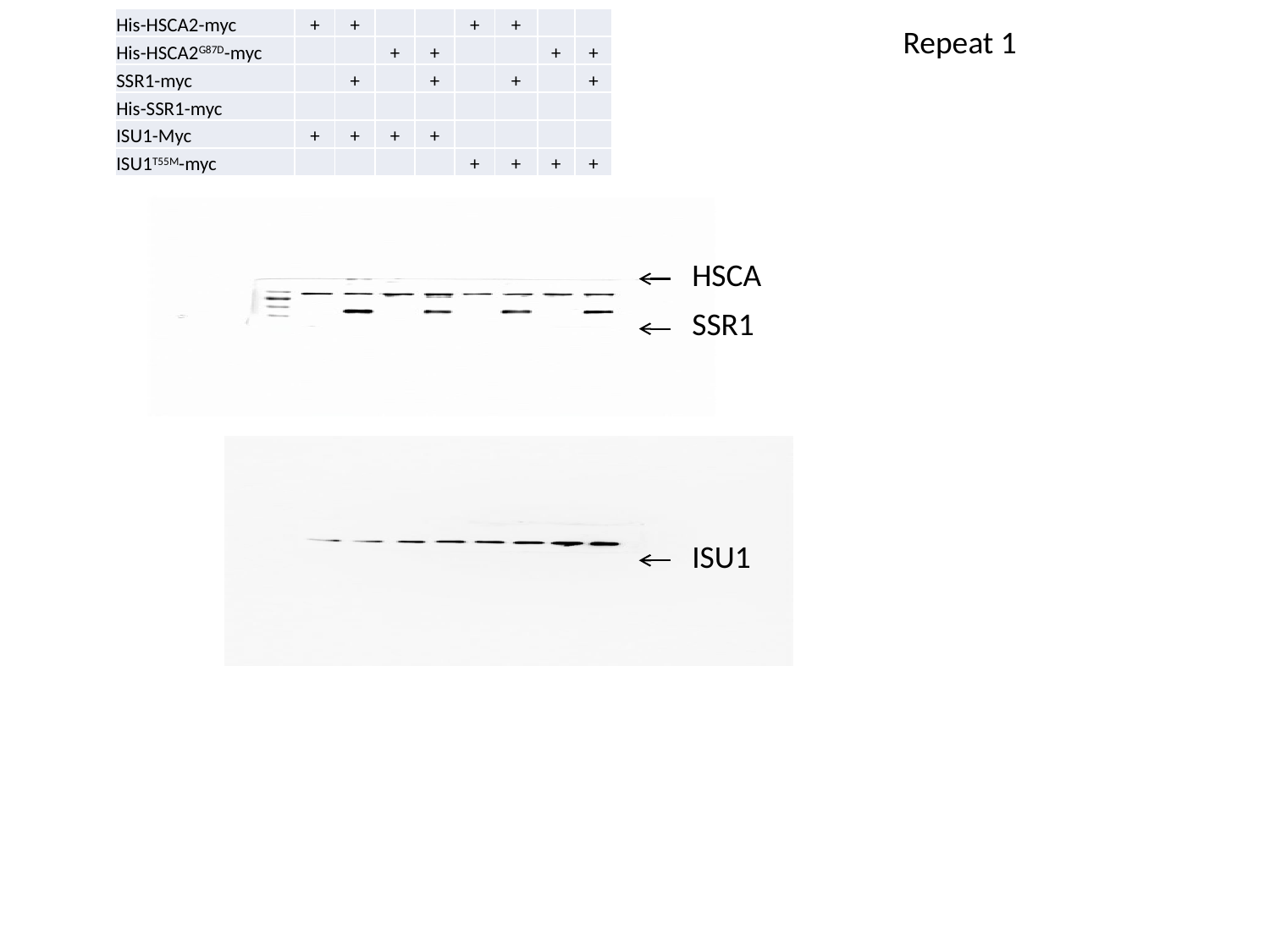

| His-HSCA2-myc | + | + | | | + | + | | |
| --- | --- | --- | --- | --- | --- | --- | --- | --- |
| His-HSCA2G87D-myc | | | + | + | | | + | + |
| SSR1-myc | | + | | + | | + | | + |
| His-SSR1-myc | | | | | | | | |
| ISU1-Myc | + | + | + | + | | | | |
| ISU1T55M-myc | | | | | + | + | + | + |
Repeat 1
HSCA
SSR1
ISU1

## Slide 5
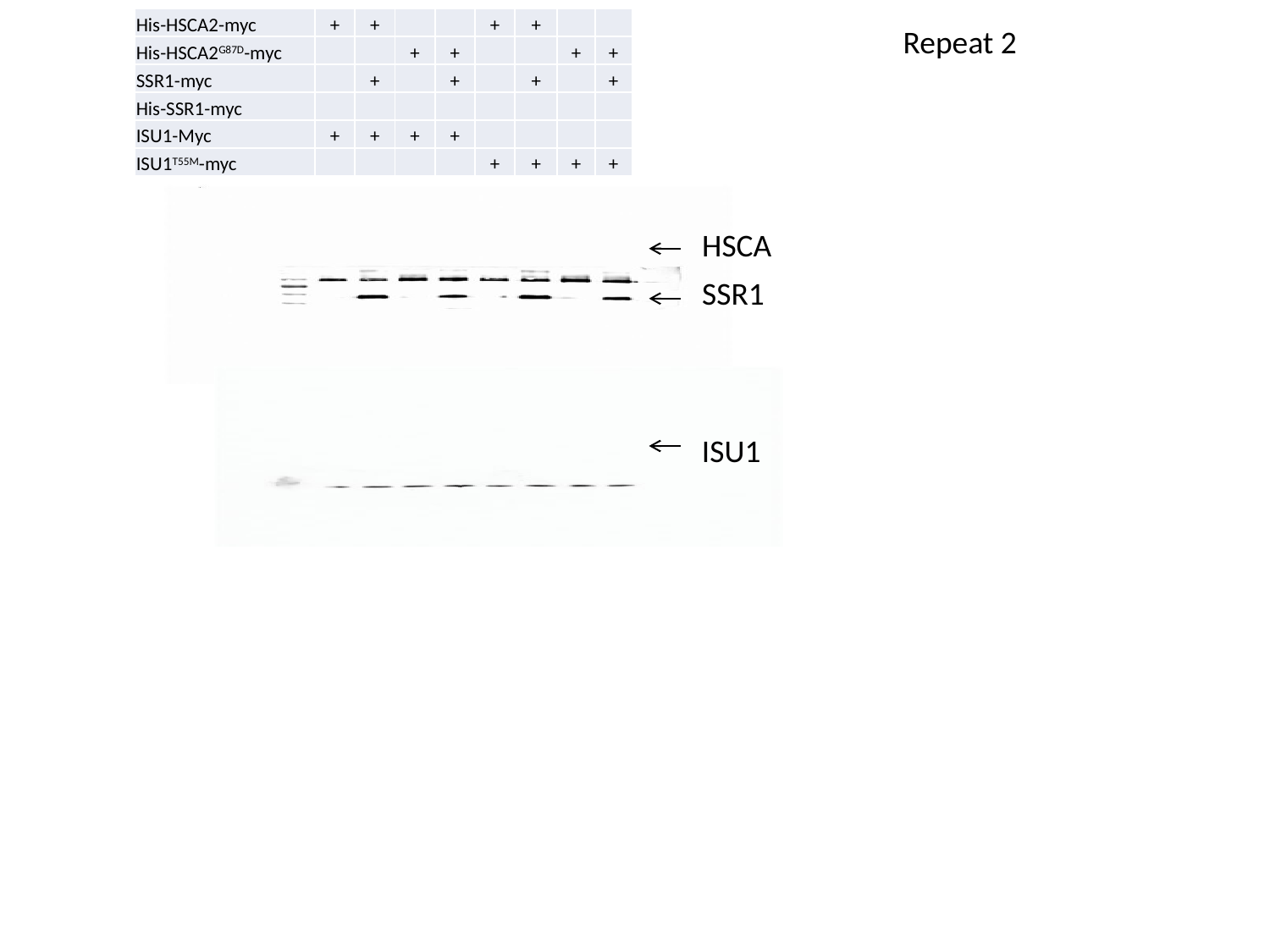

| His-HSCA2-myc | + | + | | | + | + | | |
| --- | --- | --- | --- | --- | --- | --- | --- | --- |
| His-HSCA2G87D-myc | | | + | + | | | + | + |
| SSR1-myc | | + | | + | | + | | + |
| His-SSR1-myc | | | | | | | | |
| ISU1-Myc | + | + | + | + | | | | |
| ISU1T55M-myc | | | | | + | + | + | + |
Repeat 2
HSCA
SSR1
ISU1

## Slide 6
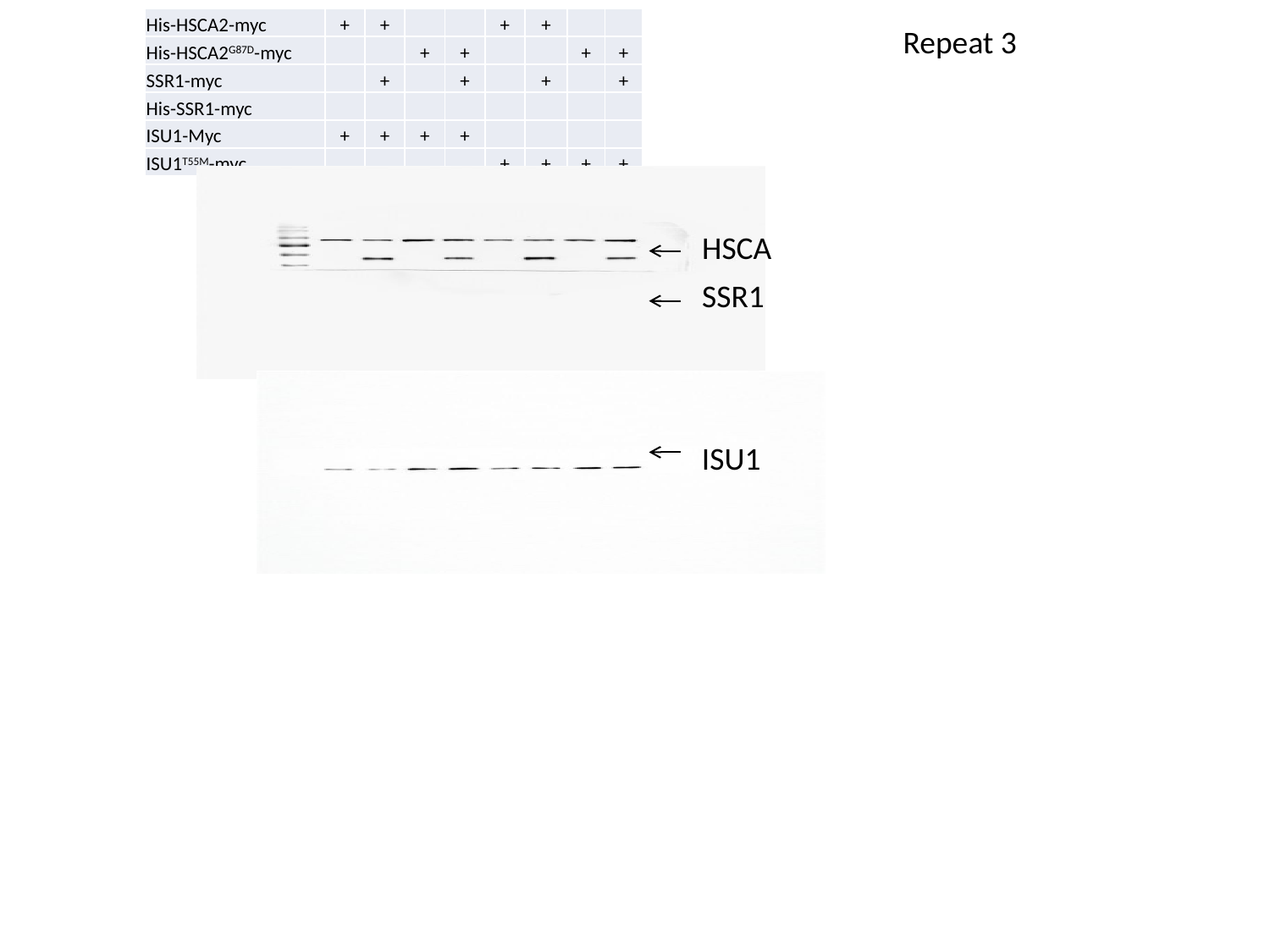

| His-HSCA2-myc | + | + | | | + | + | | |
| --- | --- | --- | --- | --- | --- | --- | --- | --- |
| His-HSCA2G87D-myc | | | + | + | | | + | + |
| SSR1-myc | | + | | + | | + | | + |
| His-SSR1-myc | | | | | | | | |
| ISU1-Myc | + | + | + | + | | | | |
| ISU1T55M-myc | | | | | + | + | + | + |
Repeat 3
HSCA
SSR1
ISU1
