# Supplementary material for: Plant-specific cochaperone SSR1 affects root elongation by modulating the mitochondrial iron-sulfur cluster assembly machinery
Source: PLoS Genet. 2025 Feb 5;21(2):e1011597. doi: 10.1371/journal.pgen.1011597 (PMC11835332; doi:10.1371/journal.pgen.1011597)
Supplement: Attachment — Submitted filename: Reply to editor and reviewers.docx [file pgen.1011597.s025.docx]

To Nicolas Rouhier, Guest Editor and

Dr. Caludia Kohler, Section editor

Dear Dr. Rouhier and Dr. Kohler,

Thank you for the message regarding our manuscript which was submitted previously as the MS# PGENETICS-D-24-00725. We really appreciate your comments and the decision for us to submit the revised version in two months. Firstly, we would like to express our sincere gratitude to the three reviewers for taking the time to review our manuscript so carefully. The constructive questions and suggestions provided by the reviewers have significantly enhanced the quality of our manuscript. Secondly, we appreciate their interest in our work and the positive comments. We have addressed each question point by point and revised the manuscript accordingly, with the changes highlighted in yellow for your convenience.

Best wishes.

Xuanjun Feng

**Response to reviewers**

**Reviewer #1:** This interesting work proposes that the plant-specific protein SSR1 acts as a molecular chaperone regulating the mitochondrial ISC machinery in response to environmental stress. The authors initially identify two suppressors of a ssr1-2 plant which is phenotypically characterized by short root length. Necessary controls have been made to verify the suppressor effects (but since I am not a plant geneticist, I cannot fully evaluate this part). The suppressors are encoded by the HSCA2 and ISU1 genes, both known to be involved in FeS protein formation. Interactions between SSR1 and the two ISC proteins are shown by BiFC (please improve quality of data presentation; I mostly see a black picture) and Co-IP studies. The latter also suggest that SSR1 supports a slightly better interaction between ISU1 and HSCA2. By introducing mutations into the LPPVK motif of ISU1 (known to be needed for HSP70 interaction from studies in bacteria and yeast), it is shown that SSR1 can stabilize their interaction to some extent, but only when these proteins are completely unable to bind to each other. The authors then tested a potential chaperone activity of SSR1 by preventing the aggregation of a model protein (CS). These studies suggest that SSR1 indeed can prevent aggregation of CS. Why is this effect not smaller when ISU1 and HSCA2 are both present since under these conditions these proteins should sequester SSR1 and thereby prevent its chaperone activity? Next, the authors find that ssr1-2 roots accumulate iron because of disturbed iron regulation (using various methods). The authors do not explain why specifically root tips take up more iron. Please, address this issue. Further, why is the iron accumulation higher around the quiescent center and the stem cell niche of the root? All the effects are again suppressed by sus1/2. Since all these data suggest that Ssr1 may play a novel role in plant mitochondrial FeS protein synthesis, the authors measure FeS protein activities (mito and cyto Aco, CI) and find them diminished in ssr1-2 and complemented by Sus1/2. Gene expression of Ssr1 is increased by proline and other measures, yet the precise reason for these effects remains obscure, and may need extra work. Finally, the authors suggest that SSR1 co-evolved with the ISC system, yet is present only in the plant lineage. Overall, the authors provide evidence for a new plant-specific member of the mitochondrial ISC machinery. The data suggest that the Ssr1 protein may act similarly to the J protein HSCB interacting with HSCAs. This aspect is not properly addressed in the manuscript and needs further attention. How does Ssr1 differ from the plant mito HSCB? The discussion of these aspects leaves the reader somewhat puzzled. A few other issues have to be resolved before publication can be recommended.

**Reply:** We would like to express our sincere gratitude to you for taking the time to review our manuscript so carefully. We appreciate your interest in our work and the positive comments. The constructive questions and suggestions have significantly enhanced the quality of our manuscript. We have addressed each question point by point and revised the manuscript accordingly, with the changes highlighted in yellow for your convenience. Regarding the few concerns, we have addressed them as following:

1. You mentioned that the BiFC results in the manuscript don't look very clear. This may be because the size of the picture in the manuscript is too small and the brightness is too low. We have adjusted the brightness of the images, and provided a number of large-size original images in the appendix materials (BiFC for Figure 2A.ppt) for review.

2. You asked “Why is this effect not smaller when ISU1 and HSCA2 are both present since under these conditions these proteins should sequester SSR1 and thereby prevent its chaperone activity?” We understand the concern, but for this in vitro chaperone activity assay, we do not expect including HSCA2 and ISU1 with SSR1 could sequester/bind significant amount of SSR1 such that the chaperone activity is compromised in the prevention of heat-induced aggregation of CS. We used purified proteins and an excess amount of SSR1 is included in the assay as shown in Figure 4C (equivalent molar ratio of SSR1, HSCA2 and ISU1, but twice the amount of CS). More importantly, as shown in Figure 4D, SSR1 has a very strong chaperone activity and even with 1/5 molar amount of SSR1, the heat-induced aggregation of CS was already dramatically inhibited, and 1:1 molar ratio almost completely inhibited CS aggregation. Therefore, when SSR1 is incubated with CS at a 2:1 ratio, CS aggregation can be completely inhibited by SSR1. In addition, our results indicate that the addition of HSCA2 or ISU1 alone effectively inhibits CS aggregation (Figure 4B). Therefore, the addition of HSCA2 or ISU1 during the SSR1 incubation with CS may also theoretically further enhance the inhibition of heat-induced aggregation of CS. Therefore, even though HSCA2 and ISU1 were able to sequester SSR1 in this experiment, we do not necessarily see that the addition of HSCA2/ISU1 promotes heat-induced aggregation of CS when SSR1 coexists with CS.

3. You mentioned “The authors do not explain why specifically root tips take up more iron.” In this manuscript, we did not indicate or claim that root tips absorb more iron. Instead, we acknowledge that the root hair growth zone of the plant root system is the main site for nutrient absorption. We focused on the root tips in this study is due to the distinct differences observed in Perls’ prussian blue staining among the root tips of different materials, while there were no significant differences in staining within the root hair zone.

4. You asked “why is the iron accumulation higher around the quiescent center and the stem cell niche of the root?” It is a very interesting question. We do not know why the iron content around the apical quiescent center is higher than that in the surrounding tissues. Therefore, we checked the literature. However, this issue does not seem to have been studied or discussed. We speculate that this may be related to the strong cell division ability and small cell size of the tissues around the quiescent center, but of course this is only speculation and has no basis. This may be a good topic to study.

5. You suggested that it is not clear enough about the possible relationship between SSR1 and HSCB. So we've rewritten this part (line 460-479).

Comments:

1. Introduction: The Eukarya-specific CIA system should be mentioned as a biogenesis system, since it is known for many years.

**Reply:** This part has been added. Line 26-27. Line 29-31.

2. Introduction, line 57: Also human cells have been widely used to study FeS protein assembly. Please add.

**Reply:** This part has been revised. Lline 38.

3. Lines 61-62: This statement is certainly wrong: “Fdx/Yfh1 was proposed to supply iron to scaffold IscU/Isu1 62 [12].” The paper does not mention CyaY (= bacterial Yfh1) and shows that Fdx plays a role in cluster fusion. Please read the literature and cite carefully.

**Reply:** thanks very much. We have carefully read the research and revised this part. Line 42-47.

4. Line 66 f: “...facilitate the release of Fe-S cluster from IscU/Isu1 to apo-proteins.” Not really correct. Both in bacteria and mitochondria a glutaredoxin is used as a trafficking factor. Please amend these sentences, and also add the DnaJ function, because that is at least as important as that of the Hsp70.

**Reply:** thanks very much. We have carefully read the research and revised this part. Line 48-55.

5. The reader is left alone with the function and localization of NFS2 and ABA3 (both are known). Please mention.

**Reply:** This sentence has been added. Line 62-63.

6. What is CK in Fig. S7B?

**Reply:** CK indicates that yeast is grown on a medium without stress. We've changed CK to Mock, so that it might make it clearer.

7. The entire manuscript should be checked for typos (there are quite a few).

**Reply:** We have done a grammatical check of the entire manuscript and revised the language again.

**Reviewer #2:** Autonomous assembly of iron-sulfur clusters in mitochondria is essential for supplying all mitochondrial iron-sulfur proteins with their respective clusters. The machinery for this cluster assembly in plants is generally closely related to the machinery in non-plant organisms like e.g. yeast and human cells with orthologous proteins for all key steps of cluster assembly. Therefore, it is very interesting to see here that a new plant-specific component of the cluster assembly machinery is proposed. In a suppressor screen conducted on the mutant ssr1-2, which is characterized by a short and swollen root phenotype, two of the isolated suppressors are mutant variants of the well-established cluster assembly core components ISU1 and HSCA2. While six (?, the number seems to vary in different parts of the text causes some confusion) other potential suppressors could not be validated (or were not characterized further), the mutants sus1 (HSCA2G87D) and sus2 (ISU1T55M) were analyzed in some more depth. These proteins are known elements of the mitochondrial iron-sulfur cluster assembly machinery and known to be involved in the release of newly assembled clusters to apoproteins. It is intriguing that the subcellular localization of these candidates matches the localization of SSR1 that was reported earlier. SSR1 contains a tetratricopeptide domain and is shown here to act as a chaperone that is capable of preventing citrate synthase from heat-induced denaturation. Based on the appearance of ISU1 and HSCA2 mutants in the suppressor screen and further presented results, SSR1 is also considered as a protein that helps establishing close contact between ISU1 and HSCA2 by which it thus may act as a chaperone or co-chaperone in FeS cluster release.

**Reply:** Thank you for taking the time to review our manuscript and put forward valuable questions and constructive suggestions, which would significantly enhance the quality of our manuscript. We have addressed each question point by point and revised the manuscript accordingly, with the changes highlighted in yellow for your convenience.

In fact, we have identified more than a dozen suppressors with different extents of suppression. Among them, *sus1* and *sus2* were determined through super-BSA analysis and transgenic complementation. For the remaining suppressors, direct sequencing on HSCA2 and ISU1 was performed and the results showed that these suppressors could be classified into eight types, designated *sus1* to *sus8*. With the exception of *sus3*, single amino acid variants of HSCA2 or ISU1 were identified in all other suppressor types. Therefore, the "eight" was used in the abstract to summarize the results, and the "a dozen" was used in the first paragraph of the result to describe the experimental process. To enhance clarity, we revised the abstract to specify 'eight kinds' instead of simply 'eight' and adjusted the initial section of the results accordingly.

Given that FeS clusters are essential for multiple processes in mitochondria and particularly for energy metabolism, the short root phenotype in ssr1 mutants could be just pleiotropic. The observed decrease in auxin transporters, the transcription factor WOX5 and the amount of auxin may thus be simply a mere secondary consequence of other defects rather than based on a causal link fo FeS cluster assembly in mitochondria. This possibility should be taken into consideration before speculating too much about the effects of FeS cluster deficiency on auxin metabolism and signalling.

**Reply:** Thank you for your comments. We completely agree with you and believe that the effects of SSR1 loss of function on auxin signaling are not direct. In fact, we have not suggested in this article that SSR1 is directly involved in regulating auxin signaling. Actually, we have mentioned in the discussion section (Page 18, Line 429) that “Therefore, it was not surprising that *ssr1-2* mutant displayed pleiotropic abnormal growth phenotype, such as short roots, brush-like roots, bushy shoots and growth retardation”. Just because we discussed extensively the effects of *ssr1-2* on the auxin signaling pathway in our last published article. Therefore, in this article we continue to look at whether suppressors can recover *ssr1-2* related defects at the microscopic level. Despite of being indirect, shortage in Fe-S supply did affect auxin biosynthesis and signaling, as being previously reported. Please refer to the references cited in line 427-447.

To prove the suspected role of SSR1 in FeS cluster supply to the respective apoproteins, activities of complex I in the respiratory chain, cytosolic and mitochondrial aconitases as well as malate dehydrogenase were measured. The provided description of the respective methods is very superficial and by far insufficient for others to repeat the experiments. E.g. the identity of the used kits is not provided and now information on the exact parameter that was measured is given. For proper evaluation of the presented data it is also necessary to show the raw data in supplemental figures rather than presenting only a bar chart for enzyme activities. The reason for this is that at least the measurement of aconitase activities is often prone to artefacts and even more so when the activity for isoforms in different subcellular compartments are to be measured. The authors measured the expected differences and effects similar to what has been described in the past. This may not be completely wrong, but it does not fully match with more recent data from Moseler et al., 2021 and Ströher & Millar, 2016 (both Plant Physiology) who both independently showed that the prime consequence of defects in FeS cluster transfer is actually in protein lipoylation with the respective metabolic consequences. The observed differences in the mitochondrial membrane potential might be a highly pleiotropic effect and thus not sufficient to support the presumed function of SSR1. It is also not clear why technically ambitious isolation of intact mitochondria was done rather than using conventional stains that label mitochondria in live cells dependent on their membrane potential. Furthermore, it is shown that the abundance of SDH2 in ssr1-2 decreases (and partially recovers with expression of sus1 and sus2). Given that aconitase is well known to become instable without FeS clusters it would have been great to show data on the abundance of aconitases as a complement for the activity measurements.

**Reply:** Thank you for your valuable questions and suggestions, which have significantly improved this manuscript. We have checked again the two articles you mentioned, which focused on the function of GRX15 in Arabidopsis thaliana. These studies found that GRX15 primarily influences the activity of lipoyl-dependent proteins. Ströher et al. reported that impaired GRX15 function did not significantly affect CI and Aconitase activity in gel assays; however, we noted that the standard error in the CI activity assay was large. In our study, we measured CI and Aconitase enzyme activities using kits, and our results indicated that the loss of SSR1 function significantly reduced the activity of both enzymes. GRX15 has been identified as an important protein for mitochondrial 2Fe-2S carry in Arabidopsis, and your findings suggest that the loss of GRX15 function primarily affects lipoyl-dependent proteins without impacting the enzymatic activity of CI and Aconitase. However, it is important to note that these results should not lead us to conclude that the impaired function of the mitochondrial iron-sulfur cluster synthesis machinery only affects lipoyl-dependent proteins, excluding the activities of CI and Aconitase. Similarly, while we suggest that SSR1 may be primarily involved in the release of iron-sulfur clusters from the ISU scaffold, this does not mean that the *ssr1-2* mutant will behave the same as the *grx15* mutant, i.e., does not affect the enzyme activity of CI and ACO. Therefore, our findings, which demonstrate that SSR1 dysfunction affects the activity of CI and ACO, do not contradict your conclusions. In addition, it is easy to find other reports showing that the enzyme activity of CI and Aconitase is affected in mutants of ISC machine-related genes (Xu et al, 2009, Dual localized AtHscB involved in iron sulfur protein biogenesis in Arabidopsis. PLoS One 4: e7662; Leaden et al, 2016, Altered levels of AtHSCB disrupts iron translocation from roots to shoots. Plant Mol Biol 92: 613-628; Voisine et al, 2001, Jac1, a mitochondrial J-type chaperone, is involved in the biogenesis of Fe/S clusters in Saccharomyces cerevisiae. Proc Natl Acad Sci U S A 98: 1483–1488; Adam et al, 2006, The Nfs1 interacting protein Isd11 has an essential role in Fe-S cluster biogenesis in mitochondria. The EMBO Journal 25: 174-183).

The information of enzyme activity assay kits was displayed in the Methods section (line 646-647 in the previous manuscript). Since the instructions that come with the kit contain detailed procedures, we have not repeated them in this article. To make this part clearer, we've rewritten this part of the method. In addition, we have added the original measured values corresponding to the histogram as appendix materials (Values of the enzyme activity for Figure 6.xls) for review. If you do not agree with the results of the ACO enzyme activity assay, we can discuss the possibility of omitting this section from our findings, as we believe it will not impact the main conclusions of our study.

We use flow cytometry to analyze the membrane potential of mitochondria, so we need to extract mitochondria. As mentioned in the method, crude mitochondria were used for this experiment, so the process is not very cumbersome. This method analyzes a large number of mitochondria at one time, and the results are more accurate than those observed using conventional staining in live cell.

You suggested that we measure the protein level of aconitase. In fact, we indeed have tried to measure the protein level of aconitase, but the commercial antibody we purchased did not work well and could not detect the target band specifically, so we did not continue to detect aconitase at the protein level.

Protein-protein interactions for ISU1, HSCA2 and SSR1 led to the hypothesis that SSR1 is involved in FeS cluster transfer from ISU1 to recipient proteins. The principle idea of an involvement of another chaperone or co-chaperone is not completely new as other chaperones are involved in this process in yeast and human cells. This is also indicated in the text. The finding that point mutations in either ISU1 or HSCA2 can at least partially compensate for the loss of SSR1 is novel and highly interesting. However, for better mechanistic understanding it would be helpful to further explore the effects of the respective mutations on the protein-proteins interactions in more details. Why exactly would the mutation T55M in ISU1 improve its interaction with HSCA2? The same question applies to G87D in HSCA2. Would structural modelling allow to deduce the respective impacts on the interactions?

**Reply:** Thanks very much. This suggestion makes a lot of sense. We used AlphaFold to predict the three-dimensional structure of the HSCA2^G87D^ and ISU1^T55M^ proteins, and further analyzed their protein-protein interactions. However, the prediction results were subpar. The pTM score of 0.67 for the HSCA2-ISU1 interaction indicates that the overall folding of the heterodimer may resemble the real structure. In contrast, the ipTM value of 0.63 indicates low reliability regarding the predicted relative positions of the interacting proteins. Notably, previous studies have identified the LPPVK sequence in ISU1 as a critical site for HSCA2 binding; however, this LPPVK region does not resided at the interaction interface with HSCA2, underscoring the misalignment in the predicted relative orientations of the two proteins. In the cartoons, we label HSCA2 in blue, ISU1 in green, LPPVK amino acid sequence in gray, and Gly87 of HSCA2 and Thr55 of ISU1 in red. Given these inaccuracies in the predicted interaction results, it is not feasible to compare the effects of amino acid variations on the interaction using this method. Nonetheless, we have included the complete results of our predictions as appendix materials (Prediction_Protein_interaction.zip) for your review.

If SSR1 indeed acts as a chaperone and apparently does interact with citrate synthase as the first tested candidate protein already, it cannot be excluded that it also binds to multiple other proteins as well. This ought to be critically assessed in the discussion. Similarly, it also should be discussed that TPR domain-containing proteins have been reported earlier to interact with Hsp70 and help them as co-chaperones in their function.

**Reply:** We fully agree with you that SSR1 has the potential to interact with other proteins. We prefer to refer to SSR1 as a chaperone rather than a co-chaperone, as SSR1 alone can exhibit strong chaperone activity, even far exceeding that of HSCA2. However, in the process of SSR1-assisted binding of HSCA2 to ISU1, it seems more appropriate to call SSR1 a cochaperone. Therefore, as you suggested, in the title of this article we refer to SSR1 as cochaperone, and we have added the discussion about this part. Line 471-479.

Additional points:

l. 60: To avoid confusion with other forms of FeS clusters, it should be considered to explicitly mention [2Fe-2S] clusters here (and elsewhere in the text).

**Reply:** It has been revised.

l. 89: ‘TPR’ needs an explanation on first mention

**Reply:** It has been added.

l. 94-94: These two sentences appear partially redundant.

**Reply:** It has been revised.

l. 101-102: The very same information was provided a few lines earlier in the Introduction already.

**Reply:** This sentence has been removed from the results section.

l. 105: here ‘a dozen identified suppressors’ are indicated, while the Abstract refers to only eight suppressors are mentioned (l. 26). Please clarify!

**Reply:** In fact, we have identified more than a dozen suppressors with different extents of suppression. Among them, *sus1* and *sus2* were determined through super-BSA analysis and transgenic complementation. For the remaining suppressors, direct sequencing on HSCA2 and ISU1 was performed and the results showed that these suppressors could be classified into eight types, designated *sus1* to *sus8*. With the exception of *sus3*, single amino acid variants of HSCA2 or ISU1 were identified in all other suppressor types. Therefore, the "eight" was used in the abstract to summarize the results, and the "a dozen" was used in the first paragraph of the result to describe the experimental process. To enhance clarity, we revised the abstract to specify 'eight kinds' instead of simply 'eight' and adjusted the initial section of the results accordingly.

l. 109-116; Fig. 1B: The effect of the ssr1-2 mutation on auxin transport and signalling is very likely highly pleiotropic (as discussed in l. 439-443) and may be merely related to recovery of root growth in the suppressor mutants rather than functionally linked to the mutation. These observations are at best complementary and could be transferred to the supplement without losing any key message. The discussion of a possible decrease in aldehyde oxidase activity due to deficiency in supply of FeS clusters would need a solid experimental base to avoid too much speculation. In this context, see also general comments above on prime metabolic effects of deficiencies in FeS cluster supply.

**Reply:** Thank you for your comments. We completely agree with you and believe that the effects of SSR1 loss of function on auxin signaling are not direct. In fact, we have not suggested in this article that SSR1 is directly involved in regulating auxin signaling. Actually, we have mentioned in the discussion section (Page 18, Line 429) that “Therefore, it was not surprising that *ssr1-2* mutant displayed pleiotropic abnormal growth phenotype, such as short roots, brush-like roots, bushy shoots and growth retardation”. Just because we discussed extensively the effects of *ssr1-2* on the auxin signaling pathway in our last published article. Therefore, in this article we continue to look at whether suppressors can recover *ssr1-2* related defects at the microscopic level. Despite of being indirect, shortage in Fe-S supply did affect auxin biosynthesis and signaling, as being previously reported. Please refer to the references cited in line 427-447. We think this part of the results is also important, so we hope to put it in the main figure rather supplemental figure.

In addition, we have removed the discussion of aldehyde oxidase as you suggested.

l. 128-129; l. 541: The supplementary tables are not available for download and thus the screening for candidate mutations cannot be assessed. It remains unclear what else beyond “genes of most interest” (based on functional annotation and prediction of subcellular localization) appeared in the initial screen.

**Reply:** We did select candidate genes as outlined in the methodology, but the process required significant effort and encountered some challenges. As mentioned in the first sentence of the fourth paragraph of the results, we selected a total of 10 candidate genes for transgenic validation for both *sus1* and *sus2*. For *sus1*, we identified 5 candidate genes for validation and successfully identified the target genes. In contrast, we initially selected only 2 candidate genes for *sus2*; unfortunately, neither of these turned out to be the actual gene of interest. To address this, we selected 3 additional genes, and fortunately, among them, we were able to identify the true target gene.

To enhance clarity in this section, we have added some information. Line 533-535.

l. 161: The phrase ‘controlling root development’ is misleading. Obviously, the deletion of SSR1 does have a severe root phenotype but this does not imply a direct function of SSR1 in control of root growth. The phenotype very likely is just pleiotropic and no surprise if basic metabolic functions like energy supply are compromised. The term ‘control’ thus should be avoided.

**Reply:** Thank you for the explanation. The description of the full text has been revised in accordance with your suggestion. Line 149.

l. 201; Fig. 2A: The presented data are not fully convincing because the fluorescence is rather weak and the labelling pattern for mitochondria does not seem typical in terms of organelle shape and number. It would be much better if a maximum projection of multiple individual sections was presented. nVenus and cCFP may well reconstitute, but the reconstituted protein is certainly not a GFP as indicated in the figure. It would be more appropriate to label this rather e.g. as ‘reconstituted FP’

**Reply:** You mentioned that the BiFC results in the manuscript don't look very clear. This may be because the size of the picture in the manuscript is too small and the brightness is too low. We have adjusted the brightness of the images, and provided a number of large-size original images in the appendix materials (BiFC for Figure 2A.ppt) for review.

The annotations in Fig. 2A have been revised as you suggested.

l. 379: ‘ed’?

**Reply:** it has been deleted.

l. 384-386: Induction of SSR1 under multiple stress conditions may be more likely related to a broad chaperone function rather than to its apparent role in FeS assembly. This should be discussed.

**Reply:** We agree with you that perhaps many chaperone proteins are induced under various stresses. Here we just want to state that *SSR1* is induced by multiple stresses, suggesting that it may be important for environmental adaptation. Combined with our findings---- *SSR1* is involved in regulating the function of ISC and ISC itself is susceptible to stress, so we point out in the discussion that *SSR1* plays an important role in helping ISC adapt to stress. In addition, our previous studies (references 28 and 29) and this study (line 369-374) showed that mutants with weakened or lost *SSR1* function are sensitive to stress. Therefore, we believe that such an inference is obvious and not excessive.

l. 404, Fig. S7A: a counterstain for mitochondria is missing.

**Reply:** we have added the new data with mitochondrial markers (Mito Tracker).

**Reviewer #3:** The manuscript describes the molecular function of SHORT AND SWOLLEN ROOT1 (SSR1) and its association with the mitochondrial iron-sulfur cluster machinery.

A genetic screen is used to identify suppressors of one of the mutations (ssr1-2) of SSR1. Two suppressors of the short growth phenotype, linked to the ssr1-2 mutation, are found, located in HSCA2 and ISU1. The association between SSR1 and HSCA2 and ISU1 is demonstrated in vivo and in vitro. In addition, the role of SSR1 as a chaperone is investigated. It is also shown that the 2 suppressors restore the mitochondrial defects previously identified in the ssr1 mutants.

The origin of SSR1 is discussed.

**Reply:** Thank you for taking the time to review our manuscript and put forward valuable questions and constructive suggestions, which would significantly enhance the quality of our manuscript. We have addressed each question point by point and revised the manuscript accordingly, with the changes highlighted in yellow for your convenience.

1) The interaction between SSR1 and the mitochondrial Fe-S machinery has never been described so far and is clearly supported by experimental data. However, some of them could be more convincing.

ex:

- the BiFC images are very weak. Is it linked to a weak expression of the two constructs? How many protoplasts were examined?

**Reply:** The transformation efficiency of protoplasts is relatively high, and we have observed fluorescence in a significant number of cells; however, the intensity of the fluorescence is quite weak. Despite repeating this experiment multiple times, the fluorescence signal remains consistently low. In contrast, we have utilized these two vectors in other experiments where the expression efficiency was notably high. We speculate that the inherent characteristics of the protein and its interaction patterns may contribute to the weak signal (see the next discussion for BN-PAGE). Nonetheless, the results clearly demonstrate that protein-protein interactions in the mitochondria are authentic and reliable. You mentioned that the BiFC results in the manuscript don't look very clear. This may be because the size of the picture in the manuscript is too small and the brightness is too low. We have adjusted the brightness of the images, and provided a number of large-size original images in the appendix materials (BiFC for Figure 2A.ppt) for review.

- the explanation about the BN-PAGE could be improved. What is the expected size of the HSCA2-SSR1-ISU1 complex? Is this size compatible with the 440 kDa signal or the one below 300 kDa? Is is made in E coli or in Arabidopsis? Could the association be improved by using different concentrations of the detergent (Tween-20) or the use of another detergent? Is it feasible to make MS on these spots on the first dimension of the gel to identify other components of the 440 kDa signal? What is the meaning of the number of hours on the top of the gels?

**Reply:** In this study, HSCA2, SSR1 and ISU1 were all expressed and purified from *E. coli.* Due to the mechanism of dynamic cycling of HSCA to substrate, the stoichiometric ratio of HSCA to ISU is uncertain (Dutkiewicz et al, Methods Enzymol, 2017, 595:161-184). However, it is clear that if the complex can be detected, its size should be at least greater than 160 kD, and if the HSCB of *E. coli* is also in the complex, its size should be at least greater than 180 kD. Although the BN-PAGE experiment is not essential in this study, we want to see if there is a tripartite complex in HSCA2-SSR1-ISU1. In previous reports, HSCA-HSCB-ISU complex formation is very inefficient because the canonical molecular mechanism of the HSP70 substrate-binding cycle (Dutkiewicz et al, Methods Enzymol, 2017, 595:161-184). In this study, although the signal of HSCA2-SSR1-ISU1 tripartite complex is clearly observed at approximately 400 kD, the signal of ISU1 is very weak. It is also consistent with the canonical molecular mechanism of the HSP70 substrate-binding cycle. The number of hours on the top of the gels represent the electrophoresis time of BN-PAGE.

- would it be possible to make structural prediction about the interactions (alphafold) and what would be the scores of these interactions, using wild type and mutant versions of HSCA2 and ISU1?

**Reply:** Thanks very much. This suggestion makes a lot of sense. We used AlphaFold to predict the three-dimensional structure of the HSCA2^G87D^ and ISU1^T55M^ proteins, and further analyzed their protein-protein interactions. However, the prediction results were subpar. The pTM score of 0.67 for the HSCA2-ISU1 interaction indicates that the overall folding of the heterodimer may resemble the real structure. In contrast, the ipTM value of 0.63 indicates low reliability regarding the predicted relative positions of the interacting proteins. Notably, previous studies have identified the LPPVK sequence in ISU1 as a critical site for HSCA2 binding; however, this LPPVK region does not resided at the interaction interface with HSCA2, underscoring the misalignment in the predicted relative orientations of the two proteins. In the cartoons, we label HSCA2 in blue, ISU1 in green, LPPVK amino acid sequence in gray, and Gly87 of HSCA2 and Thr55 of ISU1 in red. Given these inaccuracies in the predicted interaction results, it is not feasible to compare the effects of amino acid variations on the interaction using this method. Nonetheless, we have included the complete results of our predictions as appendix materials (Prediction_Protein_interaction.zip) for your review.

2) the phenotype of the ssr1-1 and -2 mutants could be presented in the introduction. This would help to understand what is new or not about the physiological role of SSR1 described in the result section.

**Reply:** This information has been added. Line 74-79.

3) What is the definition of a 'novel' gene. TPR proteins is a large family. Could additional info could be brought?

**Reply:** Given that the function of *SSR1* in the synthesis of iron-sulfur clusters has not been reported, we called it a "novel" gene in the previous manuscript (line390). Since you didn't think this was appropriate, we revised it.

Additional comments

- Could the authors comment about the semi-dominant inheritance of SU2?

**Reply:** This is an interesting and challenging question. I would like to share my perspective, and I welcome any criticism or corrections if my views are inappropriate. This is an interesting and challenging question. Based on our result, these two suppressor mutations are both gain-of-function mutations and are, therefore, dominant. However, in case of ISU1, one copy of ISU1^T55M^ may not be sufficient to totally suppress short-root phenotype of *ssr1-2* (haplo-insufficiency). In another word, in the absence of SSR1, when ISU1 and ISU1^T55M^ co-exist (the situation in heterozygous lines), both ISU1 and ISU1^T55M^ can participate in the assembly of Fe-S clusters. However, the Fe-S clusters on wild-type ISU1 may not be released as efficiently. Therefore, one copy of ISU1^T55M^ will not interact with sufficient HSCA2 and release sufficient Fe-S cluster, conferring semi-dominance phenotype. In case of HSCA1, one copy of HSCA2^G87D^ is sufficient to totally suppress the phenotype of *ssr1-2*. In another word, enough interaction with and then release of Fe-S from ISU1 could be achieved by one copy of HSCA2^G87D^. In the absence of SSR1, when HSCA2 and HSCA2^G87D^ exist at the same time, only HSCA2^G87D^ can functionally interact with ISU1 and function normally. As a result, *sus1* showed complete dominant phenotype.

- iron content: measurements against g DW or TOC would be more reliable. Are there other metals (S?, Zn) that show different contents?

**Reply:** Thank you. We agree with you, but the dry weight of the sample in this experiment is too small to measure. The contents of other metals have been added as Supplemental Table S5.

- a supplemental file with the position of the suppressor mutations for both HSCA2 and ISU1 could be added.

**Reply:** We guess your suggestion is to specify the location of the two genes on the chromosomes, right? You can get this information in Table S2 and Table S3.

- Line 520; incomplete unit

**Reply:** it has been revised.

- transcriptomics data (Fig5A) should be deposited in a public repository.

**Reply:** Transcriptomic data (Fig5A) was provided in Table S4.

- Fig6B: comment about the decrease of SDH2.

**Reply:** Fe-S cluster functions in maintaining the proper 3D structure and/or enzymatic function activity of Fe-S proteins. We speculate that this may be due to an insufficient supply of iron-sulfur clusters, resulting in the instability of SDH2 without binding iron-sulfur clusters. This is just our speculation, and there is no basis for it. If you have any other explanations, please let us know.
