# Supplementary material for: Plant-specific cochaperone SSR1 affects root elongation by modulating the mitochondrial iron-sulfur cluster assembly machinery
Source: PLoS Genet. 2025 Feb 5;21(2):e1011597. doi: 10.1371/journal.pgen.1011597 (PMC11835332; doi:10.1371/journal.pgen.1011597)
Supplement: Attachment — Submitted filename: Response_to_Reviewers_20250104.docx [file pgen.1011597.s026.docx]

**Response to reviewers**

We appreciate all three reviewers’ careful reading and comments. As to remaining concerns, we are trying our best to further clarify as indicated below, and at the same time, we revised and rearranged some sentences in the manuscript to make them clear.

Reviewer's Responses to Questions

Reviewer #1: I have checked the response of the authors to my concerns. Here is my evaluation:
1. Improved, but still borderline quality of the pictures.

R1: Thanks for your comment that the picture quality is improved. The lower quality as seen in the reviewing PDF document is likely because all figures were pre-imbedded into Word document and then converted to PDF file. All the main and supplementary figures have been uploaded separately as high resolution .tif files and they should appear as the high quality images in final publication if accepted.
2. The authors provide a lengthy explanation for my concern. One may believe this or not. Apparently, the issue is more complex than appreciated.

R2: Thank you for the comment, and as you pointed out, the function of SSR1 may be more complicated than what we currently observed. In the previous response, we attempted to explain that the in vitro biochemical assays may not essentially reproduce the exact condition in vivo. As shown in Fig 4D, SSR1 has a very strong tendency to protect CS from heat-induced aggregation and when it is used in excessive amount (Fig 4B and 4C), the effect of ISU and HSCA2 sequester is minimal and not obviously observed. But this effect could be more biologically significant in vivo when SSR1 is generally expressed at a much lower level. Anyhow, we think it would be more appropriate to conduct in-depth analyses in the future to further tackle the molecular mechanism of SSR1 and learn how it is involved in the regulation of Fe-S cluster biogenesis together with HSCA2 and/or ISU1 at the molecular level.

3. The authors’ answer (“we did not indicate or claim that root tips absorb more iron.”) is incorrect: In two parts of the old and new manuscript, the authors mention the “iron accumulation in root tips”: Abstract, line 30, and lanes 325ff (“eight times more”, old manuscript) or lane 346f (“had higher iron content in the tips”, new manuscript). Why was this text changed and the quantitation removed? This point needs further careful reassessment.

R3: We believe there is some misunderstanding regarding the wording. “Accordingly, ssr1-2 displayed increased iron accumulation in root tips and altered expression level of genes responsive to iron deficiency.” and “ssr1-2 had higher iron content in the tips and leaves than the wild type.” In these two sentences, we meant that ssr1-2 mutants have greater iron content in the root tips compared to the wild type. In our previous response letter, we attempted to claim that the root tips are probably not the primary tissue for iron absorption (as indicated by “we did not indicate or claim that root tips absorb more iron”). We revised the original sentence, “ssr1-2 had more than eight times more iron in the tips than the wild type” to “ssr1-2 had higher iron content in the tips and leaves than the wild type.” as in line 354 in the newly revised manuscript, because we have added the content data in leaves.
4. I accept that not much is known about this topic. I did not get, however, if the authors have added any statement addressing this aspect to the manuscript.

R4: Thanks, we have added two sentences at the end of the second paragraphs in the discussion section as “Notably, ssr1-2 mutants accumulated higher levels of iron in their root tips. The increased iron deposition may be detrimental to the maintenance of the quiescent center and stem cell niche, as the excess ferrous iron may trigger the Fenton reaction, leading to oxidative stress and damage to cellular structures.” (lines 499-503)

5. Relationship between SSR1 and HSCB: Do the authors refer to lines 506-516 (the authors’ callout lines 460-479 are incorrect)? This part is a valuable addition, despite its vague statement. I am sure that the reader is still puzzled in what the role SSR1 is performing (chaperone or now co-chaperone?) and how its function compares to that of HSCB.

R5: We apologize for referring the wrong line numbers mentioned in the previous reply. In the assays of heat-induced aggregation of citrate synthase, SSR1 clearly displayed the function of a chaperone. Based on this result, we initially refer SSR1 as a chaperone rather than a co-chaperone. However, since SSR1 interacts with HSCA1 and ISU1 and also by comparing the role of HSCB in the HSCA functional cycle (generally speaking J-domain protein as a cochaperone of HSP70 family protein), it is possible that SSR1 only partially overlap with HSCB for its function. We indicate this in the discussion section particularly in lines 519-522.

Comments:
1. Resolved (but the mentioned lines are wrong; also the lines mentioned in the following points; hence it is difficult to judge the responses).

R1: We apologize for the incorrect line numbers mentioned in the first round of reply. It is Line 60.

2. Resolved (despite the wrong line callout), even though I would have preferred the mentioning of the major species studied.

R2: Thank for your comment and it is line 71.
3. Slightly improved only. Still incorrect parts: i) New evidence accumulated over the past decade does not confirm a role of FXN in iron transfer to ISCU. The statement “frataxin/Yfh1/cyaY are proposed to be iron donors” should either be deleted or indicated that this view was refuted by studies in the last decade. ii) The statement “transferred to recipient apo-proteins (such as glutaredoxin Grx5)” should be changed because Grx5 is not a recipient FeS protein (like aconitase). Rather, it is a cluster transfer protein. iii) In lanes 95f, a sentence from my initial review erroneously entered the manuscript (“The reader is left alone with the function and localization of NFS2 and ABA3 (both are known”.). Please remove, and explain the localization and function of NFS2 (plastid and SufS-like) and ABA3 (cytosol and MoCo synthesis).
4. Comment included in point 3.
5. Comment included in point 3.

R3-R5: Thank you for the meticulous review and patient suggestions. We have revised this part in lines 77-82.

6. Resolved.

R6: Thanks.
7. There are still many cases where singular and plural “s” are wrong.

R7: We have carefully read trough and revised in many places as we can catch.

Overall, the authors have improved their manuscript. However, as mentioned above, there are essential parts that have to be carefully re-addressed before I can recommend publication.

**Reviewer #2**: The manuscript has been revised according to comments provided in the first round of reviewing. With these revisions some but not all shortcomings have been fixed.
You argue that the expression of auxin-related geens and the translational resonse of SSR1 to environmental stress is important to the story. I see you point that this links to your earlier publications on the characterization of the mutant. It is certainly a nice confirmation that the suppressor mutants also reactivate the expression of auxin-related genes. Yet, the effects are merely pleiotropic and don't help much to understand the function of SSR1, but rather distract the reader's attention from the core message.
R1：Thank you for your comment. We agree that the analysis on auxin related genes is not the core message of the work. However, we feel it provides one critical piece of evidence showing the true suppressor phenotype. The finding of impaired auxin transport related genes in ssr1 mutant is a key message in the previous work (Zhang et al, 2015). As you pointed out, and as we indicated in the manuscript, ssr1 mutant caused a pleiotropic phenotype. SSR1 is involved in auxin transport, in the iron-sulfur cluster biogenesis, in proline resistance, and probably others, We think that including the data have the benefit in one way to show the complexity of the SSR1 function.

The description of isolated suppressors and particularly the number of these repressors has revised. Yet, it is still confusing when in the abstract it is stated that 'eight kinds' suppressors have been isolated and in the first part of the Results section it is said that a dozen suppressors have been isolated (it is noted that in the rebuttal it is now still claimed ‘more than a dozen’). If eight of these 12 suppressors are designated sus1 to sus8, what is the designation of the remaining four suppressors? If these additional suppressors beyond sus1 and sus2 are considered important to be mentioned here, the respective identities should be revealed together with some reasoning for not following them further at this point. In the Abstract the reader is now referred to 'eight kinds of suppressors'. The phrase 'eight kinds of suppressors' suggest that these are different. Later, in the Results section, it turns out that seven out of these eight are mutants of either HSCA2 (2) or ISU1 (5), which I would count as two kinds of mutants. It is not obvious why you not simply focus on these seven mutants. You can still indicate that you have isolated other mutants but concentrate further detailed analysis on these seven mutants (or in fact one representative of each) because the large number of mutants in just two genes known to be involved in the same physiological process is highly intriguing.
R2: We appreciate you comments and understand the confusing points. To clarify, we have revised the manuscript and also included a separate supplemental Table S4 to show all the identified suppressor lines. Lines 25-26, lines 165-168.

Additional points:
It should be considered mentioning in the manuscript that attempts for structural modelling of SSR1, IU1 and HSCA2 including their interactions has been attempted but did not provide conclusive results.
R：Thanks, and yes, we have added this part in lines 245-251.

According to your response letter, the two sentences in lines 94-96 with largely redundant information have been revised. This statement is not correct as the very same sentences are still in the text, now in lines 115-117.
R: Thanks. The sentence “Its homolog could not be found in organisms of other kingdoms.” Has been deleted.

A number of line numbers mentioned in the rebuttal do no match the respective line numbers in the manuscript text.
R：We apologize for the incorrect citation of line numbers and will pay more attention in the future.

The brightness of the images in Fig. 2A have been adjusted for clarity. Such image modification procedures, even if done only for carity, should be indicated in the legend. The annotation 'FP' for the reconstituted protein is certainly more appropriate than 'GFP in the earlier text version. Yet, 'FP' should be explained in the legend.
R: Thanks, more information has been added in figure legend.

l.48: To make the Abstract text consitent with the title and the Discussion, SSR1 should be called a cochaperone here.
R: it has been revised.
l.95-96: Not clear to me what this sentence is meant to say. Delete or rephrase.
R：it has been deleted.
l.165-171: The text should be revised according to the comments provided above already; 'five additional types of mutants does not make sense because they apparently all are mutants of just two genes.
R: We provided a new supplemental Table S4 to summarize all the 14 originally identified suppressors although they are finally categorized into 8 groups with some suppressors sharing the same point mutations. At the same time, we have rewritten the corresponding text to make it clearer. Lines 165-168.

l.464: The exact number of Fe-S proteins is not known because there are still new proteins being dicovered as cluster coordinating. The total number, however, will almost certainly be much closer to 100 than 'hundreds' as stated here. The number in mitochondria, which are considered here, is much lower. Thus it would be sensible to tone this down a little.
R: Thank you for your meticulous review and patient suggestions. It has been revised.

l.509: 'We do not believe this is likely.' Delete this sentence here and discuss the data first. Draw a conclusion at the very end.
R：Thanks. it has been deleted. Line 519

l.517: 'stress-adapting' should be 'stress adaptation'

R：Thanks. it has been revised.

**Reviewer #3**: The manuscript has been improved following revision by the authors.
However, there are still some points that could be clarified.
Line 95-96: to be completed.
R: This sentence was mistakenly included in the reviewer's suggestion and has been removed from the revised manuscript.

Transcriptomics data. Providing a Table is fine but, in addition, raw data should be deposited on a public repository (NCBI, …). Raw data can be made accessible to the public only upon acceptance of the manuscript. An accession number should be provided in the manuscript.
R: Thanks for the comment and suggestion. However, we just want to acknowledge that the transcriptomic analysis was performed in 2015 right after we published the previous work in 2015 (Zhang et al, 2015, Plant J. 83: 582). The original raw data were saved in the facility server, and we only maintained the analyzed differentially expressed gene list. Unfortunately, the raw data were deleted from the facility serve as they only kept the customer data for five years. We didn’t back up the raw data because of its large size and because it was not a mandatory requirement by many journals at the time of our analysis. We hope the current analyzed data is sufficient for the understanding of the work.

Semi-dominance: I’m fine with the explanation. Fig S1 could be completed (panel D) in support to the explanation provided concerning the interaction between the WT and the mutated copies of ISU1 and HCSA2 in the presence and in the absence of SRR1.
R: Thanks. Based on our result (mainly from Figure 1D), these two suppressor mutations are both gain-of-function mutations and are, therefore, dominant. However, in case of ISU1, one copy of ISU1^T55M^ may not be sufficient to totally suppress short-root phenotype of *ssr1-2* (haplo-insufficiency). In another word, in the absence of SSR1, when ISU1 and ISU1^T55M^ co-exist (the situation in heterozygous lines), both ISU1 and ISU1^T55M^ can participate in the assembly of Fe-S clusters. However, the Fe-S clusters on wild-type ISU1 may not be released as efficiently. Therefore, one copy of ISU1^T55M^ will not interact with sufficient HSCA2 and release sufficient Fe-S cluster, conferring semi-dominance phenotype. In case of HSCA1, one copy of HSCA2^G87D^ is sufficient to totally suppress the phenotype of *ssr1-2*. In another word, enough interaction with and then release of Fe-S from ISU1 could be achieved by one copy of HSCA2^G87D^. In the absence of SSR1, when HSCA2 and HSCA2^G87D^ exist at the same time, only HSCA2^G87D^ can functionally interact with ISU1 and function normally. As a result, *sus1* showed complete dominant phenotype. Therefore, Figure 1D may be what you're looking for.

Concerning the discussion, there would be 2 roles for SRR1: one described here in the ISC machinery, and one in the response to osmotic stress and proline treatment. How these two roles could be combined in a single protein? Could they be linked to the structure of SRR1? TPR proteins may be composed of different domains (for example when they play a role in RNA edition). Is it the case for SRR1?

R: Thanks. We believe you are referring to PPR proteins, which are usually involved in RNA editing, while TPR motifs primarily interact with other proteins rather than nucleic acids. We speculate that reactive oxygen species (ROS) may be the key to connecting the two roles of SSR1 that you mentioned, as we discussed in this manuscript (lines 532-540). In our two previous publications (1. Han et al, 2021, SSR1 is involved in maintaining the function of mitochondria electron transport chain and iron homeostasis upon proline treatment in Arabidopsis. J Plant Physiol. 2. Lv et al, 2011, Proline accumulation is inhibitory to Arabidopsis seedlings during heat stress. Plant Physiol), we discussed this topic as well. Excess proline leads to electron overflow in the mitochondrial electron transport chain (mETC) due to either inhibited mETC activity or increased electron supply, resulting in elevated ROS production. The ISC machinery is particularly vulnerable to inhibition by ROS.
