# Supplementary material for: Plant-specific cochaperone SSR1 affects root elongation by modulating the mitochondrial iron-sulfur cluster assembly machinery
Source: PLoS Genet. 2025 Feb 5;21(2):e1011597. doi: 10.1371/journal.pgen.1011597 (PMC11835332; doi:10.1371/journal.pgen.1011597)
Supplement: Attachment — Submitted filename: Response_to_Editor_20250124.docx [file pgen.1011597.s027.docx]

To Dr. Nicolas Rouhier, Guest Editor，

Dr. Tanja Slotte, Section editor,

Aimée Dudley and Anne Goriely, Editor-in-Chief

Dear Dr. Rouhier, Slotte, Dudley, and Goriely,

We appreciate your interest and careful review of our manuscript. Thank you for the message and the decision for us to submit the revised version regarding our manuscript MS# PGENETICS-D-24-00725_R2. As you suggested, we have removed the transcriptome-related results and revised the relevant legends and the reference numbers of the Tables.

In this revised version, the mainly revised content is shown below:

**SSR1 affects iron homeostasis**

The ISC machinery plays a key role in maintaining cellular iron homeostasis by regulating the uptake, storage, and distribution of iron [33,34]. This helps to prevent iron overload or deficiency, which can lead to oxidative stress and damage to cellular structures. Overexpression of *AtHSCB* led to higher iron accumulation in roots and lower iron content in shoots, whereas *hscb* knockdown mutants exhibited reduced iron uptake in roots but increased iron levels in shoots [35]. Perls’ prussian blue staining showed that *ssr1-2* has an unusually high iron content in the root tips (Fig 5A). Iron content in root tips and leaves was further measured using inductively coupled plasma-mass spectrometry (ICP-MS). *ssr1-2* had higher iron content in the tips and leaves than the wild type (Fig 5B, Table S5). Furthermore, the accumulation of iron around the quiescent center and the stem cell niche of the root was obviously higher in *ssr1-2* than in the wild-type (Fig 5C). As expected, *SSR1, sus1,* and *sus2* well restored the iron accumulation phenotype in the root tips and leaves of *ssr1-2* mutants (Figs 5A-C, Table S5).

The resubmitted manuscript includes 7 Figures, Supporting information with 9 Supplemental Tables, 7 Supplemental Figures, and 1 supplemental dataset, as well as a Supplemental Appendix containing resource data for the main Figures. We hope this revised version will meet your requirements. Please let us know if you feel anything is missing.

Best wishes,

Xuanjun Feng on behalf of all coauthors
